# A comparison of Oxford nanopore library strategies for bacterial genomics

**DOI:** 10.1186/s12864-023-09729-z

**Published:** 2023-10-20

**Authors:** Thomas Sauvage, Alexandre Cormier, Passerini Delphine

**Affiliations:** 1 Ifremer, MASAE Microbiologie Aliment Santé Environnement, F-44000 Nantes, France; 2 Ifremer, IRSI-SeBiMER, F-29280 Plouzané, France

**Keywords:** Hybrid, Native, Leakage, Ligation, Mapping, minION, Tagmentation, Tandem, Transposase, Replicons

## Abstract

**Background:**

Oxford nanopore Technologies (ONT) provides three main library preparation strategies to sequence bacterial genomes. These include tagmentation (TAG), ligation (LIG) and amplification (PCR). Despite ONT’s recommendations, making an informed decision for preparation choice remains difficult without a side-by-side comparison. Here, we sequenced 12 bacterial strains to examine the overall output of these strategies, including sequencing noise, barcoding efficiency and assembly quality based on mapping to curated genomes established herein.

**Results:**

Average read length ranged closely for TAG and LIG (> 5,000 bp), while being drastically smaller for PCR (< 1,100 bp). LIG produced the largest output with 33.62 Gbp vs. 11.72 Gbp for TAG and 4.79 Gbp for PCR. PCR produced the most sequencing noise with only 22.7% of reads mappable to the curated genomes, vs. 92.9% for LIG and 87.3% for TAG. Output per channel was most homogenous in LIG and most variable in PCR, while intermediate in TAG. Artifactual tandem content was most abundant in PCR (22.5%) and least in LIG and TAG (0.9% and 2.2%). Basecalling and demultiplexing of barcoded libraries resulted in ~ 20% data loss as unclassified reads and 1.5% read leakage.

**Conclusion:**

The output of LIG was best (low noise, high read numbers of long lengths), intermediate in TAG (some noise, moderate read numbers of long lengths) and less desirable in PCR (high noise, high read numbers of short lengths). Overall, users should not accept assembly results at face value without careful replicon verification, including the detection of plasmids assembled from leaked reads.

**Supplementary Information:**

The online version contains supplementary material available at 10.1186/s12864-023-09729-z.

## Background

Since the public release of the MinION™ from Oxford Nanopore Technologies (ONT) in 2014, nanopore sequencing has experienced continuous improvements in terms of read quality and data output. The company has also greatly expanded library preparation strategies in order to suit customers’ sequencing needs. Whilst this new product diversity has provided users with flexibility in sample preparation, navigating ONT’s kit panel and kit denominators can be difficult. Indeed, without detailed examination of kits’ content and workflows on ONT’s website [1], novice seeking the most appropriate library strategy for their projects may face a great deal of confusion to choose amongst existing preparation kits and adds-on « expansion» kits. Moreover, ONT’s continuous product iteration (i.e. product update following chemistry improvements) increases the listing of available kits, which requires careful checking to insure compatibility between flow cells, sequencing and expansion kits.

Three main strategies of library preparation are available for standard DNA input (400–1000 ng) (Additional file 1 Table S1). These allow for sample multiplexing with molecular indices, or « barcoding» in ONT’s jargon, via tagmentation (« Rapid Barcoding» kit), ligation (« Native barcoding» kit) and PCR amplification (« PCR barcoding»). Some workflows, such as the ‘Rapid’ tagmentation-based libraries, require little to no consumables, while the PCR-based libraries necessitate up to six additional enzymes, representing an estimated cost > 1500 € and much longer preparation time (Additional file 1 Table S1). In orientating their choices, users also need to consider the number of samples to be multiplexed, which may vary with a kit’s version iteration, allowing 12, 24 or 96 samples to be run concurrently.

While ONT provides some indications of the limitations and advantages of the different kits, their impact on flow cell output and on bacterial genome assembly remains difficult to appreciate without conducting comparative sequencing runs on the same batch of samples. ONT clearly mentions that ligation kits will generally allow the greatest output possible because generated read lengths are representative of fragments initially present in solution. By contrast, the tagmentation and amplification approaches may lower the output because they further reduce the size of fragments present in solution by « cutting» DNA strands randomly or generating amplicons that cannot exceed the processivity of the long range *Taq* (respectively). The ratio of successfully adapted vs. non-adapted molecules may also differ between these strategies and lower effective DNA concentration on the flow cell.

When comparing the output of different library preparations, the number of sequences and their cumulative length may be examined for an entire flow cell or more finely at the channel level (i.e. per channel output). Indeed, MinION flow cells harbor 512 channels that can thread DNA fragments simultaneously [2] and depending on the flow cell quality (e.g. number of pores at run start, age/shelf life) and library input type (preparation approach, fragment size and DNA concentration), channels may exhibit variable output from one another. Optimally, the number of reads threading through pores and their cumulative length should be somewhat homogeneous across channels to maximize flow cell output [2]. In previous work [3], we observed reads made of abundant tandem repeat artifact not representative of the genome, which often led to the assembly of artifactual tandem contigs. To date, the tandem output at the flow cell and channel level, and across different library preparation has not been carefully investigated.

**Fig. 1:**
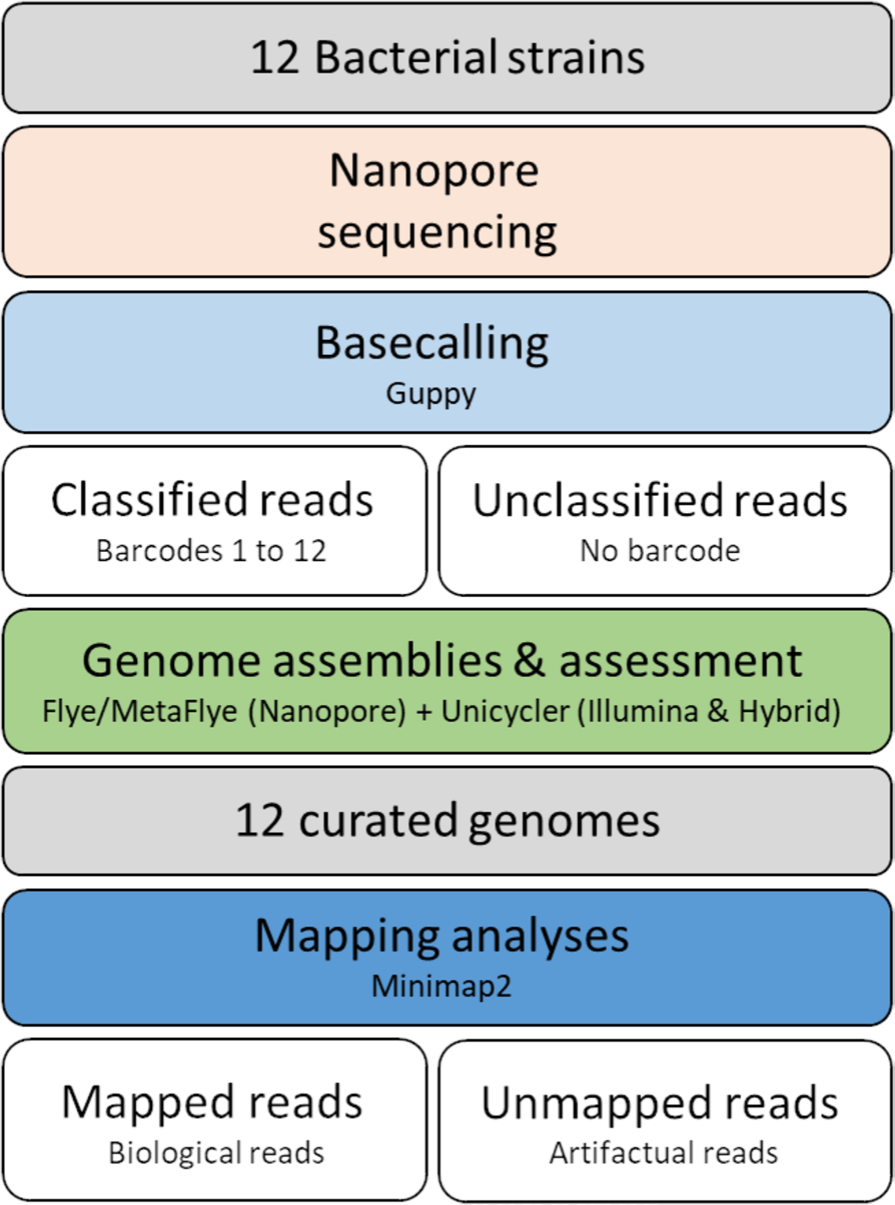
Analysis workflow and nanopore read categories. Overall analysis workflow used in the present study to obtain nanopore read categories (classified, unclassified, mapped, and unmapped) and the 12 curated genomes from the sequenced bacterial strains. Note the use of Illumina libraries for genome assembly (alone or hybrid) for comparison with nanopore assemblies toward establishing curated genomes for mapping analysis

Demultiplexing of barcoded libraries is currently conducted with ONT’s software Guppy following basecalling of the reads’ raw electronic signal into *fastQ* format. In this process, reads’ with successfully called barcodes are categorized as ‘classified’ and separated into individual folders corresponding to each barcode name (Fig. 1). On the other hand, reads whose barcodes could not be called are merged in an ‘unclassified’ folder. When a curated genome is available, reads may be mapped to assess sequencing noise (i.e. quantify artifactual and very poor quality reads) and leakage across samples (i.e. when a read’s barcode does not match the genome to which it mapped to, also known as barcode jump, tag jump, index hopping, sample-to-read-misassignment, sample bleeding or cross-talk, e.g. see [4] for a definition). Indeed, such analysis will separate mappable reads (‘mapped’), i.e. matching a genome, from non-mappable reads (‘unmapped’), i.e. not matching any of the genomes (Fig. 1) as well as confirm a read’s genomic/sample origin in order to identify leakage. In addition, mapping may allow to recover some ‘unclassified’ reads (Fig. 1) toward increasing coverage of barcoded samples that accumulated few reads, and/or to improve the assembly of complex genomes.

Here, we tested the sequencing of 12 bacterial strains (Table 1) on MinION flow cells (chemistry R9.4) with ONT’s three main library preparation strategies for genomic samples, ligation (LIG), tagmentation (TAG) and amplification (PCR). The 12 cultured strains, originating from seafood microbiome, were chosen amongst 100 strains sequenced with Illumina paired-ends (PE) (D. Passerini, unpublished) for displaying different GC content, estimated genome size and levels of fragmentation, suggesting a broad panel of genome variability (Table 1). Our main objectives were to assess the sequencing performance of these three kits (flow cell and channel output, sequencing noise and barcoding efficiency) as well as to assess the quality of resulting assemblies in a native (nanopore-only) or hybrid framework (nanopore and Illumina) compared with curated genomes established herein.

**Table 1:** Selected bacterial strains and their Illumina assembly statistics

Species (MASAE accession)	Gram	Phylum, Order, Family	Contigs	GC	N50 (bp)	Mbp
*Serratia proteamaculans* (CD3406)	-	Pseudomonadota;Enterobacterales;Yersiniaceae	96	54.8%	163,495	5.87
*Serratia proteamaculans* (EBP3064)	-	Pseudomonadota;Enterobacterales;Yersiniaceae	60	55.2%	273,789	5.46
*Serratia fonticola* (MIP2602)	-	Pseudomonadota;Enterobacterales;Yersiniaceae	124	53.6%	139,619	6.21
*Morganella morganii* (HIS2824)	-	Pseudomonadota;Enterobacterales;Morganellaceae	43	50.3%	257,021	4.14
*Hafnia paralvei* (MIP2461)	-	Pseudomonadota;Enterobacterales;Hafniaceae	68	48.0%	347,145	4.89
*Photobacterium phosphoreum* (MIP2473)	-	Pseudomonadota;Vibrionales;Vibrionaceae	73	39.5%	144,700	4.48
*Shewanella baltica* (SF1039)	-	Pseudomonadota;Alteromonadales;Shewanellaceae	88	46.3%	150,379	5.05
*Pseudomonas fluorescens* (SF1671)	-	Pseudomonadota;Pseudomonadales;Pseudomonadaceae	187	60.1%	82,654	7.58
Weeksellaceae sp. (MIP2422)	-	Bacteriodata;Flavobacteriales;Weeksellaceae	42	36.6%	158,725	3.27
*Bacillus velezensis* (SAF3325)	+	Bacillota;Bacillales;Bacillaceae	36	45.9%	326,966	4.14
*Lactococcus piscium* (SAF3333)	+	Bacillota;Lactobacillales;Streptococcaceae	68	38.5%	132,316	2.25
*Carnobacterium maltaromaticum* (SF2022)	+	Bacillota;Lactobacillales;Carnobacteriaceae	26	34.3%	591,805	3.46

Species name and MASAE collection accession for the 12 bacterial strains tested for nanopore sequencing. Phylum, order and family affiliation according to NCBI taxonomy. The number of contigs produced from Illumina assembly with Unicycler (pipeline CELIA) and resulting GC content (%), N50 (in bp), and estimated genome size (Mbp), are provided as an appreciation of genome fragmentation and putative genomic complexity. Note that with Illumina assemblies, *Pseudomonas fluorescens* was the most fragmented genome and *Carnobacterium maltaromaticum* was the most contiguous one

A caveat of our study was the impossibility to barcode samples for our ligation (LIG) kit iteration (Spring 2022, SQK-LSK110, see Additional file 1 Table S1) because ONT never released a compatible Native barcoding expansion kit for it; hence we could not examine the prospects of this type of library for assembly of individual strains and achieved barcoding efficiency. Nonetheless, in order to compare its sequencing output of ligation with that of tagmentation (TAG) and amplification (PCR) libraries, we conducted a pooled run of the 12 genomes without barcodes.

## Results

### Assemblies

Assemblies of barcoded libraries showed that the majority of bacterial chromosomes could not be assembled into circular molecules with PCR while the TAG library led to circular contigs for many of the strains regardless of assembly approach (hybrid or native, Table 2, see Additional file 3 Table S3 & S4 for further details on Flye/MetaFlye assemblies). Few exceptions were strains SF1671, SAF3325, SAF3333 and MIP2473, which required reassembly after pooling libraries PCR + TAG, or sorting of the LIG library to increase nanopore read coverage with further verification using additional assemblers (canu [5] and wtdbg2 [6]).

The assembly of circular plasmids with PCR was more successful than that of chromosomes, but TAG still comparatively resulted in more circular molecules (Table 2). Interestingly, we noted that in Flye/Metaflye assemblies, several plasmids represented concatemers (two successive copies stitched together), for which we found no evidence in the raw data via mapping. This seemed to happen more often for plasmids < 25 Kbp. Overall, the hybrid assembly with TAG performed the best for plasmids since most were complete and did not represent concatemers (see Table 2’s indices). It is to be noted that Illumina (PE) reads alone assembled several circular plasmids correctly.

Regarding chromosomes, comparison of mapped nanopore reads in the Integrated Genome Viewer (IGV) on the hybrid and natively assembled chromosomes revealed occasional missassemblies, one large inversion (in SAF3333 with hybrid PE + TAG), tandem repeat length variation (e.g. HIS2824) as well as the stitching of artifactual tandem repeats (in MIP2461 with TAG). Interestingly, Flye crashed for two strains (MIP2473 and SAF3325) even following increase memory allocation (Table 2). MetaFlye seemed more robust to this problem; nonetheless, it also crashed for some libraries or completed the assembly with abnormaly high coverage (> 1,000X for MIP2473 with the TAG library).

**Table 2.**
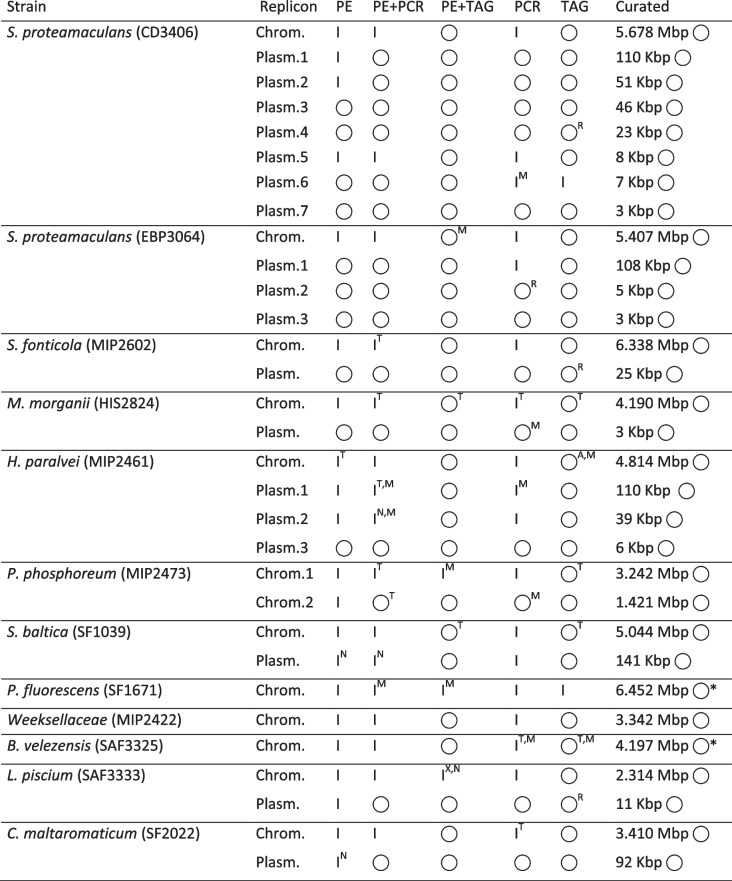
Assemblies of barcoded libraries

Results of Illumina Paired-Ends (PE), hybrid and nanopore assemblies with Unicycler (PE, PE + PCR, PE + TAG) and Flye/MetaFlye (PCR and TAG) for the barcoded libraries (no barcoding was possible for LIG, see methods). Assemblies are reported as Incomplete molecule (I) or Complete circular molecule (  ). The polished size of curated circular replicons is reported in the last column. Assembly notes are documented with the following indices: near circular molecule (N), presence of small missassemblies (M), tandem repeat length variation (T), large chromosome inversion (X), plasmid concatemer (R), presence of artifactual tandems (A). Note that the two replicons marked with an asterisk (*) (SF1671 and SAF3325) were reassembled with sorted LIG data for circularization and/or further confirmation of the chromosome scaffold. See methods and Additional file 4 Fig. S3 for further information on assembly assessment and replicon curation

**Fig. 2:**
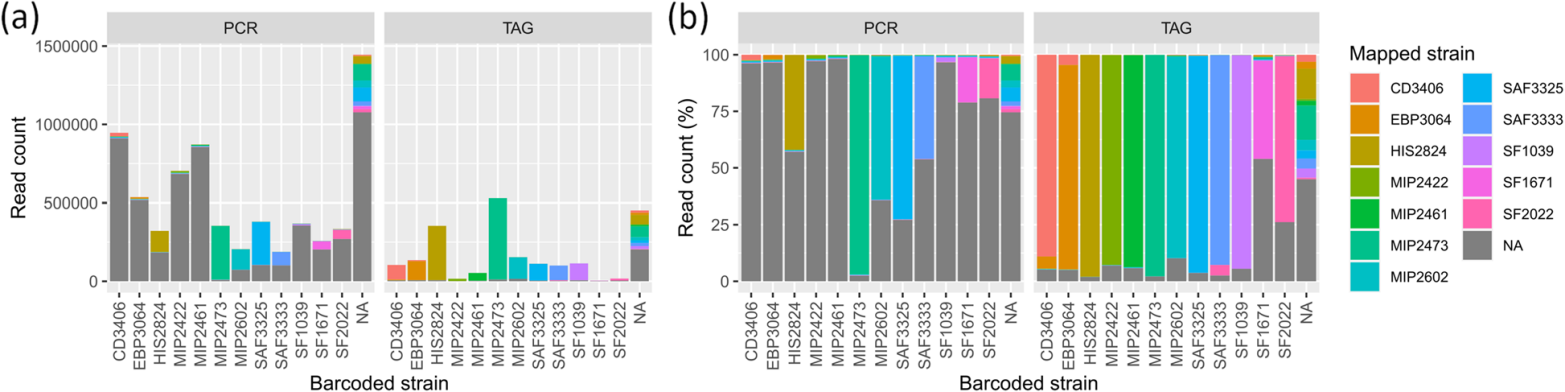
Mapped profiles per barcode and library preparation strategy. Taxonomic profile determined by mapping with Minimap2 per barcoded strain for PCR and TAG. **a** Profile in read count. **b** Profile in percentage of read count. Note the abundance of artifactual reads in the PCR runs as compared to TAG (mapped strain « NA» in the legend). Note as well the presence of leakage in TAG SAF3333 from SF2022. The initial barcode detection and classification from nanopore raw data is done with the basecaller Guppy

Finally, we found three leaked plasmids occurences in Illumina assemblies (in CD3406, MIP2422 and SF1671) that resulted from libraries run on the same Illumina flow cell at Microsynth (Balgach, Switzerland) (not shown). Logically, these leaked plasmids were also present in the hybrid assemblies from the Illumina reads. We also found one foreign circular plasmid that MetaFlye assembled from nanopore reads leaked across barcoded strains of the TAG library (i.e. plasmid assembled in SAF3333 from leaked SF2022 reads, see Fig. 2b). The assembly of unmapped reads, those not matching any of the established curated genomes herein (see methods) also further confirmed that no overlooked replicons were present (see discussion for details).

### Flow cell output

**Fig. 3:**
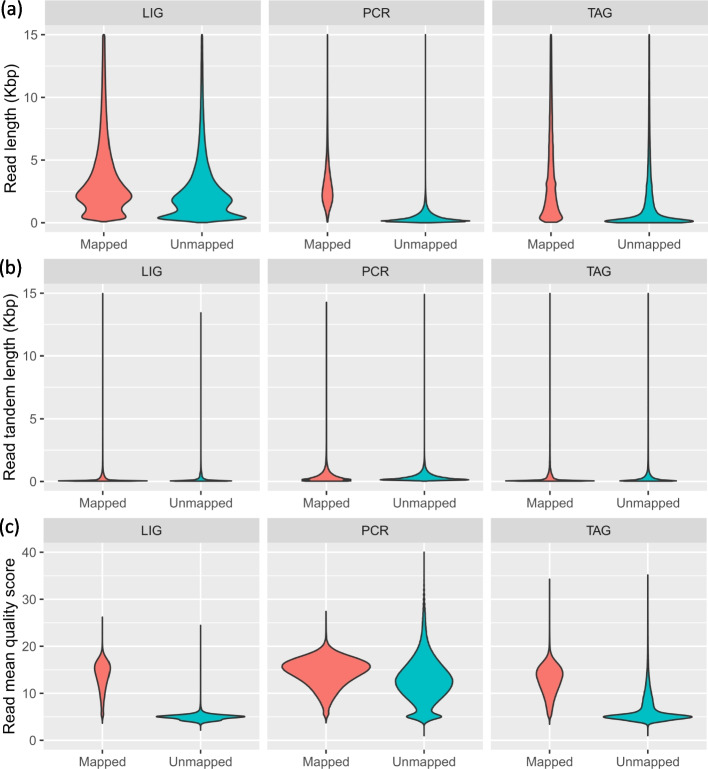
Read attributes per library preparation strategy. Vioplot depicting the distribution of read attributes per flow cell run with different library preparation strategy for mapped and unmapped reads (determined from mapping to the curated genomes established herein). **a** Read length in Kbp. **b** Within read tandem length in Kbp. **c** Read mean quality score. Note that read length and tandem length are shown up to 15 Kbp for clarity because upper tails extend beyond 100 Kbp

Library preparation strategy had an important impact on flow cell output (read number and cumulative read length, Table 3). The LIG and PCR runs produced similar read numbers (> 6.52 M and 6.92 M reads, respectively); however, their cumulative read length varied drastically (> 35.14 Gbp vs. > 7.27 Gbp) indicating much longer reads produced with LIG (see next section). Comparatively, the TAG run had much smaller read counts (> 2.15 M reads) but intermediate output (12.40 Gb) between that of LIG and PCR, also denoting the production of long reads (see next section). The majority of LIG reads (6.06 M, representing 92.9% of all reads) could be mapped to the established curated genomes, followed by TAG reads (1.88 M or 87.3%) and PCR reads (1.57 M or 22.7%). While only a small proportion of PCR reads were mappable, they made up most of the cumulative read length sequenced (4.79 Gbp out of a total of 7.27 Gbp, or 66.0%), meaning that unmapped reads were mostly very small in size (< 1,000 bp, see Fig. 3 and next section). Amongst the three library preparation strategies, PCR harbored the most reads with detected tandem content (4.33 M reads out of 6.92 M total reads, thus 62.6%) and cumulative tandem length (1.63 Gbp out of a total of 7.27 Gbp, thus 22.5%). TAG and LIG harbored much less reads containing tandem sections (0.60 M and 1.23 M, respectively, i.e. 27.7% and 18.9%) and magnitude lower cumulative tandem length than PCR (0.28 Gbp and 0.32 Gbp, respectively, representing 2.2% and 0.9%). Most of detected tandems in PCR was found within unmapped reads (Table 3).

**Table 3:** Flow cell output and tandem content per library strategy

	Reads			Tandem content		
	Mapped	Unmapped	All	Mapped	Unmapped	All
LIG	6.06 M	0.46 M	6.52 M	1.09 M	0.14 M	1.23 M
	33.62 Gbp	1.52 Gbp	35.14 Gbp	0.29 Gbp	0.03 Gbp	0.32 Gbp
PCR	1.57 M	5.34 M	6.92 M	0.54 M	3.79 M	4.33 M
	4.79 Gbp	2.47 Gbp	7.27 Gbp	0.22 Gbp	1.41 Gbp	1.63 Gbp
TAG	1.88 M	0.27 M	2.15 M	0.51 M	0.09 M	0.60 M
	11.72 Gbp	0.68 Gbp	12.40 Gbp	0.20 Gbp	0.08 Gbp	0.28 Gbp

Number of reads in millions (M), cumulative read length and cumulative tandem repeat length in Giga base pair (Gbp) for all reads from library runs LIG, PCR and TAG. The breakdown of all reads is also detailed as mapped and unmapped reads to curated genomes. Note the large number of unmapped reads (very low quality or artifactual reads) and their tandem content in the PCR strategy

### Channel output

**Fig. 4:**
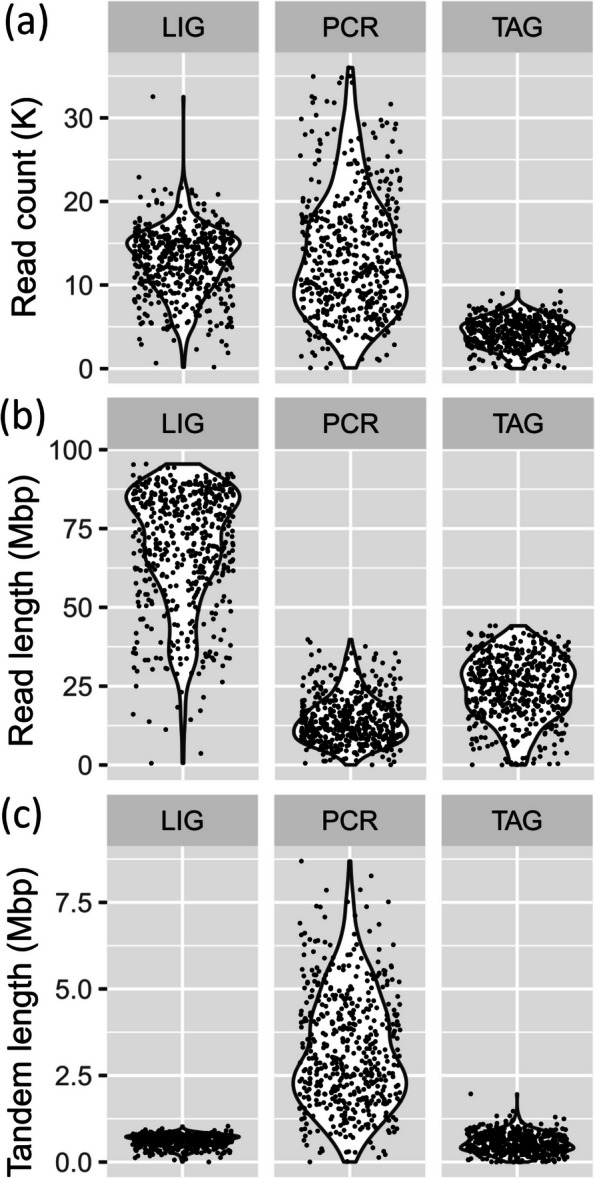
Channel output per library preparation strategy. **a** Total read count per channel in K (i.e. × 1000) per channel. **b** Total read length generated per channel in Mbp. **c** Total tandem length generated per channel in Mbp

The average number of reads per channel was close for LIG and PCR (~ 13,000 reads generated per channel) and much higher than for TAG (~ 4000 reads). However, the standard deviation was much larger in the PCR run as compared to LIG and TAG (± 7,207 bp vs. ± 3,927 bp and ± 1,703 bp, respectively, Table 4), indicating a more important variations in the number of reads threading per channel for that run. This increased variability in the PCR run is clearly visible in Fig. 4a with multiple channels producing > 30,000 reads, when other channels produced < 2,000 reads. Overall, LIG channels produced more homogeneous read counts per channel (~ 20,000 reads) perhaps reflecting better DNA saturation, i.e. better pore occupancy due to a more homogeneous library with longer reads and a more optimal DNA concentration.

**Table 4:** Mean channel output per run and tandem content

	Reads			Tandem content		
	Mapped	Unmapped	All	Mapped	Unmapped	All
LIG	11,898 ± 4,014	903 ± 1,866	12,801 ± 3,927	2,141 ± 631	281 ± 590	2,422 ± 739
	66.06 Mbp ± 19.67	2.98 Mbp ± 6.84	69.04 Mbp ± 18.79	0.56 Mbp ± 0.17	0.06 Mbp ± 0.09	0.62 Mbp ± 0.17
PCR	3,088 ± 1,817	10,499 ± 5,553	13,587 ± 7,207	1,062 ± 562	7,438 ± 3,865	8,500 ± 4,044
	9.42 Mbp ± 5.51	4.86 Mbp ± 2.93	14.28 Mbp ± 7.53	0.43 Mbp ± 0.23	2.77 Mbp ± 1.44	3.21 Mbp ± 1.66
TAG	3,692 ± 1,580	537 ± 807	4,221 ± 1,703	1,003 ± 440	168 ± 291	1,169 ± 501
	23.07 Mbp ± 9.93	1.34 Mbp ± 3.09	24.37 Mbp ± 9.95	0.38 Mbp ± 0.19	0.16 Mbp ± 0.16	0.55 Mbp ± 0.28

Average number of reads, cumulative read length and cumulative tandem length in Million base pair (Mbp) for all reads from library runs LIG, PCR and TAG. The breakdown of all reads is also detailed as mapped and unmapped reads to curated genomes. Averages are reported with their standard deviation to appreciate output variation per channel and libraries

The average cumulative read length per channels mirrorred that of the entire flow cell, being maximum in LIG, intermediate in TAG, and the least in PCR (~ 69 Mbp, ~ 24 Mbp and ~ 14 Mbp, respectively, Fig. 4b and Table 4). In LIG, a few channels produced up to > 90 Mbp (Fig. 4b). Comparatively, in PCR and TAG, channels produced maxima of ~ 40 Mbp.

Finally, regarding the cumulative tandem length generated per channel, the PCR library exhibited drastically larger values and variability than LIG and TAG (total of 3.21 Mbp vs. 0.62 Mbp and 0.55 Mbp, Table 4, Fig. 4c). In PCR, some channels produced > 7.5 Mbp of tandem while it did not exceed 1.2 Mbp in LIG and TAG, to the exception of a couple channels in the latter. The number of reads with detected tandem in PCR was also drastically larger than for LIG and TAG (Table 4). As previously determined, tandems were very abundant in unmapped reads of the PCR library (Table 4).

### Read properties

The average read length for all reads was in close range for the TAG and LIG library (both > 5,000 bp) (Table 5 and Fig. 3a). PCR reads were drastically smaller with an average < 1,100 bp. Looking at read length distribution for mapped vs. unmapped reads showed that the latter was in general always much shorter (e.g. average of 3,050 bp vs. 463 bp for PCR). Moreover, some of these unmapped reads were extremely long, as seen from the upper tail of the vioplot (Fig. 3a) and the reported maximum read size in Table 5. For instance, the PCR library largest (maximum) read length measured was 973,820 bp, which clearly represents an artifactual read considering the limited processivity of long range *Taq* not exceeding 30–40 kbp. Computing the actual base pair aligned by Minimap2 (reported in parenthesis as ‘matched’ in the first column of Table 5), that is the actual length of a read mapping to the curated genome, showed much lower numbers than the reported mapped read full length, i.e. 151,880 bp for the LIG library, 127,754 bp for the TAG library and 27,226 bp for the PCR library (vs. 227 Kbp, 394 Kbp and 488 Kbp, respectively, Table 5; note that unmapped read cannot be reported with a base pair match length since they do not match the curated genomes). The latter value of 27 Kbp for PCR is in much greater agreement with maximum long range *Taq* processivity.

**Table 5:** Mean and maximum length of reads (bp) and detected tandems per library strategy

	Read length			Tandem length		
	Mapped (Matched)	Unmapped	All	Mapped	Unmapped	All
LIG	5,552 (5,082)	3,305	5,394	47	69	49
	227,602 (151,880)	417,193	417,193	111,016	267,933	267,933
PCR	3,050 (2,569)	463	1,051	141	264	236
	488,457 (27,226)	973,820	973,820	461,075	653,416	653,416
TAG	6,250 (5,844)	2,498	5,773	104	301	129
	394,082 (127,754)	723,502	723,502	337,155	703,325	703,325

Mean and maximum length of reads determined for all mapped and unmapped reads including their tandem repeat length. Matched value in parenthesis represents the actual length (number of nucleotides) that could be mapped to curated genome as reported by Minimap2. Mapped and matched lengths differ because part of a mapped read may only align partially due to sequencing artifact within the read. Unmapped reads do not have matched values because no alignment is produced by Minimap2

**Fig. 5:**
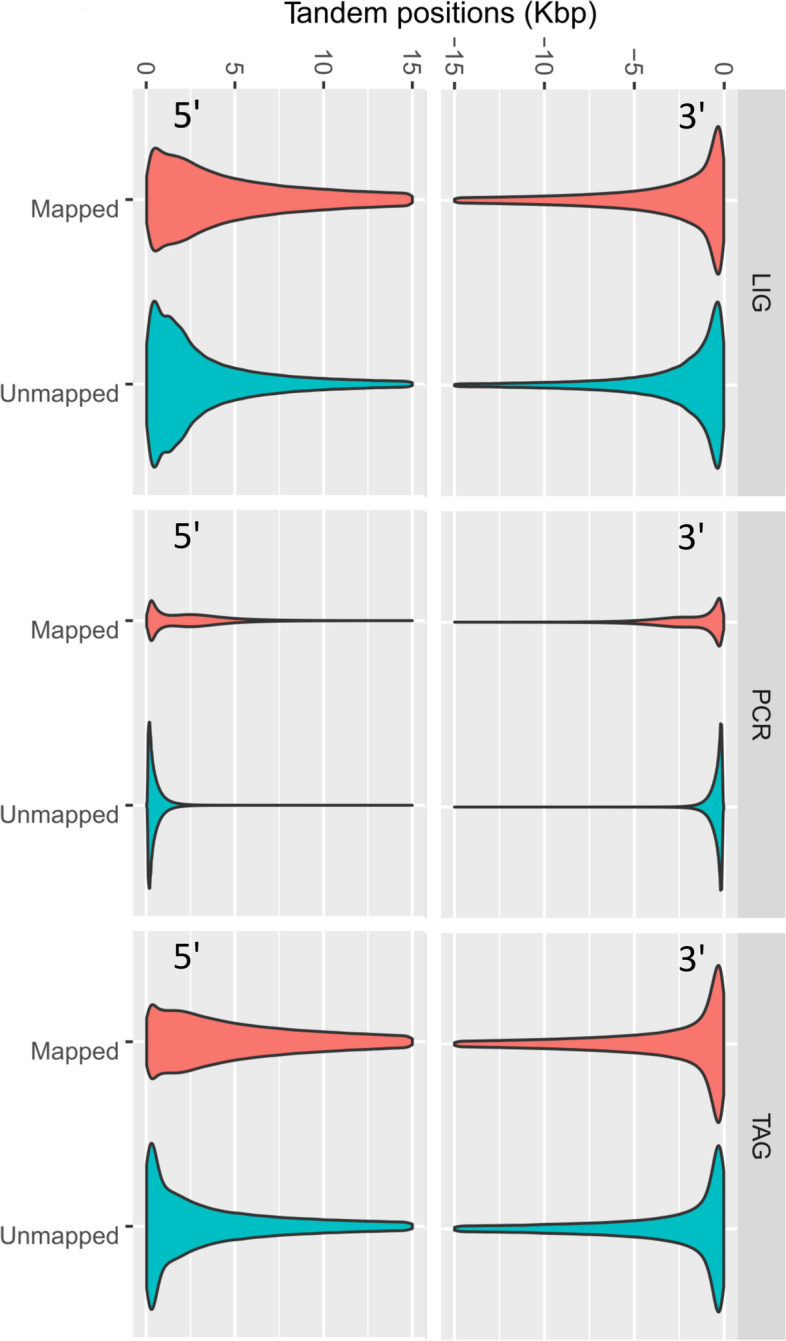
Tandem repeat distribution along reads per library preparation strategy. Location of tandem repeats along the first 15 Kbp on each side of the read (5’ and 3’ orientations represent the sequencing direction, through a nanopore, i.e. entry and exit side of a read). Tandem locations were obtained with TRF and a custom R script named TROP in order to merge overlapping tandem coordinates (see methods)

An examination of detected tandem length within reads demonstrated that for all reads, they represented a small span of the read (i.e. > 40 bp to > 230 bp in average) but that some reads accumulated extremely long continuous tandem sections (> 100 Kbp to > 700 Kbp). This was true for both mapped and unmapped reads. Overall and in average, the TAG and PCR libraries produced longer within-read tandem repeats sections than the LIG library (Fig. 3b, Table 5). The majority of tandem repeats also tended to accumulate on the first 500–2500 bp of the 5’ and 3’ side of reads (pore entry and exit according to sequencing direction, Fig. 5).

Looking at read quality in terms of mean Qscore per read showed that unmapped reads generally exhibited lower quality than mapped reads for LIG and TAG, possibly because they include more or the lower quality fail reads; however, while artifactual, some unmapped reads do have high quality (see upper tails, Fig. 3c). By contrast, and surprisingly, the Qscore of mapped and unmapped reads in the PCR library were nearly equivalent (Fig. 3c), possibly because they result from amplification artifacts that sequenced correctly through pores. By contrast, artifactual reads and LIG and TAG likely results from the sequencing process itself. Looking at mapped read quality in terms of reads’ percent identity reported by Minimap2 (not shown), showed that PCR reads had lower quality than LIG and TAG (mean of 84.3% vs. 90.3% and 91.3%, respectively, and median of 92.9%, 95.4% and 96.0%, respectively; note that percent identity values cannot be determined for unmapped reads).

### Barcoding efficiency

Based on Guppy’s basecalling and demultiplexing for the PCR or TAG library (no barcoding was possible for LIG, see introduction and methods), we observed that about > 20% of reads produced per flow cell were lost as unclassified (no barcode recovered, Additional file 4 Table S6). Within barcoded reads (Table 6), that is classified reads, mapping revealed that > 94% were correctly assigned in the TAG library, but only > 20% for PCR. Indeed, despite having a barcode, the majority of classified reads in PCR were unmapped, > 78% vs. < 5% in TAG (see Table 6 and the size of grey bars in Fig. 2). The remainder, < 1.5% of reads in each of the PCR or TAG library, represented leaked reads across barcoded samples (i.e. a read whose barcode name did not match the genome to which it mapped to) (Table 6). Leaked reads were particularly visible in the TAG library (Fig. 2b) for SAF3333 (presence of SF2022, see previous section about foreign plasmid in nanopore assembly) and within the two *Serratia proteamaculans* CD3406 and EBP3064; nonetheless, this appeared to be caused by a small proportion of short nanopore reads mapping equally to these two close strains which share 91.1% chromosome identity. Per barcode (i.e. strain), leakage ranged from 0.7% to 1.6% in PCR and 0.1% to 5.8% in TAG (Additional file 4 Table S7). Interestingly, in the PCR library, the proportion of unmapped reads varied widely per sample, some with > 95% (e.g. CD3406, EBP3064, MIP2422, and MIP2461), while others exhibited very little (2.5% for MIP2473, see Fig. 2 and Additional file 4 Table S7). Likewise, in TAG, two samples exhibited significant number of unmapped reads, > 25% and > 50% for SF1671 and SF2022, while others were relatively low (most exhibited < 7%). Looking at all of the above in terms of cumulative length, revealed putative barcode leakage more clearly for the PCR library (Additional file 4 Fig. S3b), although, this may be confounded by the equal mapping of short nanopore reads to multiple genomes rather than true leakage.

**Table 6:** Classified reads content

	Mapped		Unmapped	All
	Correct	Leaked		
PCR	1.13 M	0.07 M	4.27 M	5.47 M
	3.16 Gbp	0.26 Gbp	1.48 Gbp	4.91 Gbp
TAG	1.61 M	0.02 M	0.07 M	1.70 M
	9.98 Gbp	0.07 Gbp	0.02 Gbp	10.07 Gbp

Content of reads classified by Guppy (i.e. reads with a barcode) in millions of reads (M) and cumulative length in Giga base pair (Gbp). Classified reads were sorted via mapping to the curated genomes to determine reads that were correctly assigned (the read barcode is in agreement with the genome it mapped to), those representing barcode leakage (the read barcode is not in agreement with the genome it mapped to) or those unmapped (reads with very low quality or artifactual). See Addtional file 4 for further details on barcoding statistics

## Discussion

### Curated assemblies

We sought to establish curated genomes to allow the mapping of reads in order to quantify unusable data (unmapped reads) and barcoded efficiency (classified, unclassified and misclassified read output). To determine the best assemblies, we looked for congruence between hybrid and native assemblies using individual (PCR or TAG) or combined libraries (PCR + TAG) augmented from filtered LIG reads for complex genomes (following their sorting via stringent mapping) (Additional file 3 Table S3). We also occasionally used different assemblers (canu and wtdbg2) in order to gain further confidence (not shown). In this process, human intervention remains mandatory because tools to automate the checking of different contig assembly solutions still requires bioinformatic development [7]. Here, it is impossible to report on all of the genomic variations observed; nonetheless, in complicated cases, the use of multiple tools and long-read mapping visualization in IGV helped determine which contig to validate as best replicon (e.g. a smooth vs. broken mapped read profile). Decisions were always taken in the most conservative way and in light of potential known pitfalls in nanopore assemblies (i.e. presence of long repeats, phage excision/integration polymorphism, see Fig. 2 in [7]). Ultimately, to verify that we indeed obtained all circular replicons present in the data generated, we also assembled all unmapped nanopore reads with Flye/MetaFlye (artifactual reads not matching curated genomes or matching with very poor quality). This resulted in the assembly of contigs made of artifactual tandem repeats (up to > 270 Kbp length), chimeric contigs matching curated genomes at very low percent identity, or short contigs (< 3,000 bp) matching repetitive regions, but no additional circular replicons were found (not shown).

### Libraries output

The PCR library produced the smallest read length (~ 1,000 bp in average vs. > 5,000 bp for TAG and LIG) with maximum read length < 30 Kbp due to long range *Taq* processivity. It also resulted in the most abundant artifactual reads (> 75% of reads were unmapped, Table 3) and the widest output variability per channel, indicating suboptimal sequencing performance with some pores threading too many reads and others very few (see standard deviation in comparison to the mean, Table 4, and the widespread read count per channel, Fig. 4a). Likewise, the TAG library showed wide output variation per channel, perhaps due to the wide distribution in fragment lengths that is produced by the tagmentation approach (i.e. without a defined peak, Fig. 3a, and up to > 120 Kbp, Table 5) or subotpimal concentration of adapted molecules. By comparison, the LIG library exhibited a defined peak of abundance at 2,500 bp (Fig. 3a, maximum read length > 150 Kbp, Table 5), which perhaps explain its much better sequencing efficiency as seen by its narrower channel output (Fig. 3a). It also had very few channels producing little data (i.e. very few data points close to 0 values on Fig. 4) and produced significantly higher cumulative sequence length output (Fig. 4b, > 35 Gbp, Table 3).

Toward improving the output of PCR libraries, it is possible that fragmenting DNA prior to long range amplification would lead to more homogeneous pool of amplicons and reduced sequencing artifact. Here, we were careful of depleting genomic DNA of low molecular fragments using 0.4X magnetic bead wash prior to library preparation, but sequencing still resulted in overabundant short fragments in the PCR library. Since neither TAG nor LIG libraries suffered from such an acute overabundance of short fragments, we suspect that the problem originates from the *Taq* activity rather than faulty manipulation of the DNA during library preparation. Overall, the TAG library output appeared intermediate (some noise, moderate read numbers of long lengths) between the best results from LIG (low noise, high read numbers of long lengths) and those less desirable from PCR (high noise, and high read numbers of short lengths).

### Hybrid vs. Native

Assemblies of native libraries with PCR or TAG differed in terms of completion of plasmids and chromosomes into circular contigs (compare columns PE + PCR vs. PE + TAG, and columns PCR vs. TAG, Table 2). In general, hybrid (Unicycler) or nanopore (Flye/MetaFlye) assemblies conducted with the TAG libraries resulted in more circularized contigs and less misassemblies. This was most likely due to the much greater length of TAG reads. Indeed, for near equivalent mappable (i.e. usable) read numbers (1.57 M vs. 1.88 M), the TAG library provided cumulative sequencing length more than twice that of PCR (11.72 Gbp vs. 4.79 Gbp), which seemed critical to circularize contigs regardless of the assembly approach (hybrid vs. nanopore) (Tables 2 and 3). Indeed, while initially appearing as having high read counts (6.92 M, Table 3), most of the PCR library turn out to be artifactual (i.e. unmapped) with no value for genome assembly. Unfortunately, the LIG library could not be barcoded (see methods) but would probably have led to the most circular contigs in native or hybrid approach considering its read lengths similar to TAG and very abundant reads (6.06M mappable reads for LIG vs 1.88M for TAG, Table 3).

Overall, It is difficult to tell which of the hybrid (short-read-first assembly followed by scaffolding with long read) or native (long-read-only with short read polishing) approaches should be favored when both sources of data are available. A safe practice may simply be to compare both as was done here. For instances, regarding chromosomes, the nanopore approach with the TAG library succeeded to circularize MIP2473 and SAF3333, while the hybrid approach failed (Table 2). However, we also detected instances where nanopore assemblies led to misassemblies while hybrid ones were correct (the chromosome of MIP2461 and SAF3325). In the case of plasmids, the hybrid approach did seem to outperform nanopore assemblies (also noted in [7]). This is because Flye/MetaFlye tended to misassemble plasmids into concatemers (see CD3406 Plasm.4, MIP2602, and SAF3333, Table 2), a problem that others have reported prior as “doubled plasmids” [7]. Interestingly, the shorter reads of the PCR library seemed to cause less issue in this regard (Table 2). Thus, this problem may only arise for small plasmids assembled with longer reads. We also noted that Flye/MetaFlye sometimes circularized small contigs made of repeats, which users may initially consider as plasmids when browsing the “assembly_info.txt” file, but these generally did not match any biological sequences online (e.g. on GenBank, not shown). A simple mapping/BLASTing of putative plasmids on themselves allows for the detection of concatemers or repeats.

Finally, in exploring the genomic complexity of the different strains sequenced (Additional file 3 Table S5 and Illumina assembly fragmentation, Table 1), we could not pinpoint at any specific genomic attributes or taxonomic basis (Gram ±) that may be responsible for assembly issues or incongruence observed between hybrid and nanopore approaches. Nonetheless, three of the strains SF1671, SAF3325 and MIP2473 were quite complex, harboring phage for the former and the second, and the presence of two chromosomes for the latter. One of MIP2473 chromosome also cumulate a whopping 24 copies of 16S rDNA. We suspect the presence of genomic polymorphism in the form of structural variants (SVs) due to phage excision/integration for SF1671 and SAF3325 (see chromosome length difference between the TAG and the sorted LIG library assemblies, Additional file 3 Table S3).

### Barcoding efficiency

In both TAG and PCR libraries, about 80% of reads could be classified by Guppy, leaving about 20% of reads unusable as unclassified (e.g. recoverable to some extent via mapping as we did here for some samples with the LIG library) (Table 6 and Additional file 4 Table S6). We unfortunately could not test native barcoding with our LIG library to determine if the rate of data loss would be similar or perhaps lower since no expansion kit was available (see methods). Nonetheless, in checking the literature, we found rates of 16–26% loss for ligation runs using previous kit iteration (i.e. SQK-LSK109) and 21–28% loss for tagmentation runs [8], both of which range similarly with the present study. Interestingly, it would appear that the PCR library strains exhibiting numerous unmapped reads also had relatively small average read length (Additional file 4 Table S9) and possibly these samples became contaminated by small amplification by-products caused by the long range *Taq* activity. Regarding leakage, we found overall rates < 1.5% for both TAG and PCR libraries but larger variability at the sample level for TAG (up to 5.8% for TAG vs. up to 1.6% for PCR). Previously, others have reported higher overall rates of 3.8% for tagmentation and 2.9% for ligation libraries [8]. This possibly denotes improvement in basecalling/demultiplexing from recent versions of Guppy (v3.6.1 in [8] vs. v6.1.1 here).

### Tandem repeats

From our assessment of tandem stretches in the three nanopore library tested, their artifactual nature cannot be denied because (i) the tandem content varied between library preparations (LIG, TAG and PCR) for the same genomes, (ii) the maximum tandem length detected greatly exceeded that measured in the curated genomes (Table 5 vs. Additional file 3 Table S5), (iii) the tandem length observed in PCR was well-beyond the read length possibly amplified by the long range *Taq* (see values >>> 30 Kbp range in Table 5), and finally, (iv) detected tandems tended to accumulate unequally along the read, being more abundant on the edge of reads (both 5’ and 3’ according to threading orientation in the pore; this pattern was also true for mappable or unmappable reads, Fig. 5).

Among the three library kits tested, the greater tandem content was found in PCR reads, especially unmapped ones (Table 3). Determining the exact factor responsible for this pattern is beyond the scope of the study, but possibly relates to the presence of amplification by-products and/or template slippage, which may translate into erroneous tandem sequences of both short and extreme lengths (< 100 bp to 100s of Kbp), the latter of which being well-beyond the fragment length possibly generated from *Taq* (Table 5). The cumulative tandem length produced per channel in the PCR library was also extreme (Fig. 4c, Table 4). Indeed, tandem represented > 30% of the nucleotides produced and one channel even produced > 50% (values as percentages not shown but computed from data corresponding to upper dots in Fig. 4c). By contrast, TAG and LIG channels produced tandem lengths representing < 10% and < 5% of the nucleotides produced, respectively, which may be considered as normal background levels in nanopore data.

Finally, we used TRF as a classic tool for tandem detection [9] with a custom script [10]) to join tandems whose coordinates overlap. Future studies desiring to characterize tandem content could test recent software development for comparison. These include nucleotide-based detection softwares, such as TideHunter [11], NCRF [12], NanoSTR [13], mTR [14], and signal-based softwares, such as DeepRepeat [15] and WarpSTR [16], all of which are potentially computationally much faster than TRF [9]. Their use to develop trimming tools represent a potential avenue of research to remove/mask artifactual tandems from raw reads prior to assembly. Indeed, this may be important as we spotted the integration of artifactual tandem repeats on one of our chromosome assembly (MIP2461, Table 2, see indices). We hypothesize that such issue may happen when sufficient reads share the same artifactual tandem sequence on their edge (Fig. 5).

### Insights

As demonstrated by our results with different assemblers and library datasets (Table 2), users should generally not accept assembly results at face value without further checking. Indeed, for beginning researchers in nanopore (or long-read) sequencing, knowing how to characterize genomic variation between Mbp-scale contigs (i.e. chromosomes) can be daunting, particularly because no clear guidelines on how to best proceed are available. Tools like Trycycler [17] may save users a lot of time to build a consensus from multiple assembly files but also require human intervention (e.g. further checking of the read pile-up profile in IGV) and may not necessarily be useful in complicated cases as mentioned by its author [7]. Moreover, Trycycler requires conducting multiple assemblies of the data with different assemblers, or using a single assembler with multiple subsets of the data, which may only be possible when coverage is in excess to insure circularization. In our testing of additional assemblers than Flye/MetaFlye, we found that canu (with or without low coverage option) provided very reliable results but circularization may have to be further checked and that wtbg2 generally produced less precise contigs but was outstandingly fast. Users interested in a comprehensive testing of other long-read assemblers for bacterial genomics, may refer to [18] for notes on their advantages and drawbacks. Overall, we found that the assembly summary file format provided by Flye/MetaFlye is particularly convenient to quickly compare and interpret the content and completion of chromosomes and plasmids (presence of circular molecules, summarized contig length, etc.). Other long-read assemblers would probably benefit in providing similar reporting style.

The presence of genomic polymorphism in the form of structural variants (SVs) and the proportion of these variants across clones of a bacterial colony present in the DNA extract can strongly impact the assembly process (e.g. see structural heterogeneity discussion and related figures in [7]). We suspect that for two of our strains, SVs were present and responsible for Kbp-scale variation between the assembled chromosomes from different libraries (Additional file 3 Table S3 for SF1671 and SAF3325, see TAG vs. LIG). Assemblers reconstruct SVs to some extent but these contigs generally contain misassembled segments. Indeed, prior to the development of specific tools (e.g. [19, 20]), characterizing SVs required manual curation of contigs by comparison to raw nanopore reads (e.g. see SVs discovered in canu assemblies in [3]). Testing for, and characterizing SVs, should probably be part of assembly pipelines since their occurrence is likely underestimated and probably more than often responsible for incongruent assemblies between datasets and tools.

### Perspectives

In the present study, the TAG library performed relatively well to circularize most chromosomes based on the data output achieved for 12 samples and their native assembly with Flye/MetaFlye (Table 2). The LIG library definitely offered the largest output and would probably have resulted in the best assembly results (i.e. all replicons circular) thanks to increased coverage. Unfortunately, native barcoding could not be conducted (see introduction and methods). Accounting for the 3X greater output of the LIG library (Table 3), the barcoding and circularization of 24 bacterial strains (or more) on a minION flow cell is likely possible via ligation. ONT’s recent move from 12 to 24 barcodes for tagmentation and ligation kits with the development of V14 chemistry may thus be particularly convenient for multiplex bacterial genomic projects, namely with the Rapid Barcoding Kit 24 V14 (SQK-RBK114.24) and Native Barcoding Kit 24 V14 (SQK-NBD114.24), respectively. Note that with V14, the latter kit enables multiplexing via ligation without an added expansion kit. This may reflect an effort from ONT toward kit panel simplification. By contrast, the PCR strategy, which is still sold as an expansion for the standard ligation kit remains limited to a 12 barcode design (as of 30/05/2023). Considering the poor performance of this type of library (abundant noise, shorter reads) and poor assembly results (Additional file 3 Table S3), we cannot recommend this approach for genomic projects, except in cases where DNA concentration might be limiting or replicons to be assembled are small (e.g. plasmids). Future studies may seek to determine whether fragmentation of gDNA prior to amplification reduces sequencing noise to more acceptable levels that observed herein (> 75% unmapped reads).

With R9.4 flow cells, the use of Illumina data remains critical to polish final nanopore assemblies and avoid lingering insertion/deletion errors that may affect downstream gene annotation. The recent development of R10 flow cells (R10.4.1 currently), which provides increased read quality and homopolymer basecalling thanks to a longer nanopore barrel and dual reader head, has opened the way to near perfect genomes, potentially eliminating the need for Illumina short-read polishing [21]. However, the testing of such flow cells shows that the quality of assemblies does presently vary per bacterial strains and some, were far from near-perfect ([22]). According to the latter author, errors appeared linked to extremely long homopolymers or unusual methylation patterns that basecallers have not been trained for. It is to be noted that the above assessments of R10 flow cell relied so far on simplex read sequencing (i.e. single strand, previously call 1D reads) and that perhaps further errors may disappear in duplex read sequencing (i.e. complementary strand sequencing, previously called 1D^2^ reads). Further training of basecallers may also over time alleviate these issues ([22]). Overall, while perfect nanopore-only genome appear at reach, the need for Illumina data for polishing may not just yet be obsolete. Aside for their use in hybrid assembly, Illumina data can also facilitate the filtering of low quality nanopore reads based on kmer matching [23], and ultimately, polishing can help verify the quality of nanopore-only assemblies in the absence of genomic reference.

## Materials & methods

### Bacterial strains

Bacterial strains were obtained from the MASAE laboratory culture collection (Microbiologie Aliment Santé Environnement, Ifremer, Nantes, France), which was established from the exploration of seafood products’ microbiome for over 30 years. The 12 strains were selected for representing a broad panel of genomic variability (see Table 1) amongst 100 strains sequenced with Illumina paired-ends (PE) (D. Passerini, unpublished). These strains include both Gram negative and positive species, and represented overall 10 families found in three phyla (Bacillota, Bacteroidota, and Pseudomonadota). Three closely related *Serratia* species were also included, as they represent a seafood associated genus of focus in the MASAE laboratory (Microbiologie Aliment Santé Environnement, Ifremer, Nantes, France).

### DNA extraction

Glycerol stock of the 12 selected strains were grown in 10–20 mL of media for 24 h-48 h (depending on individual strain’s growth) in order to obtain large concentration of high molecular weight DNA for nanopore. Cells were pelleted by centrifugation at 5000 rpm for 10 min at 20 °C and the supernatant discarded. The cell pellet was then resuspended in EDTA at 50 mM for DNA extraction via precipitation (no column) using the Wizard® Genomic DNA Purification Kit, Promega, Madison, WI, USA) (see modified protocol in Additional file 5). DNA extracts were then washed with 0.4X magnetic beads (Mag-Bind® Total Pure NGS, OMEGA BIO-TEK, Norcross, GA, USA) to decrease low molecular weight fragments. No fragmentation of the DNA strands was performed in order to maintain maximum fragment lengths in solution. All DNA concentrations were measured with a Qubit 3.0 fluorometer (Invitrogen, Life technologies, Löhne, Germany) with an AccuGreen™ Broad Range dsDNA Quantification Kit (Biotium, Fremont, CA, USA).

### Nanopore sequencing

The DNA extracts were prepared with three different genomic library preparation strategies, each sequenced on separate MinION flow cells R9.4 (FLO-MIN106D) on a MinION MK1C. The libraries prepared included: (i) « PCR Barcoding of genomic DNA» (Ligation kit SQK-LSK110 with expansion 1–12 kit EXP-PBC001 consisting of long range PCR and sequencing adapter ligation), (ii) « Rapid Barcoding Sequencing» (kit SQK-RBK004 for tagmentation with barcoded transposome) and (iii) « Genomic DNA by ligation», which was conducted on the pool of the 12 samples without barcodes (i.e. Ligation kit SQK-LSK110 without Native barcoding expansion, which was never released by ONT for this kit iteration, i.e. only available for the earlier Ligation kit SQK-LSK109 and newer kits SQK-LSK114). The latter ligation run was primarily conducted to examine overall differences in flow cell output and for eventual bioinformatic sorting (i.e. additional long read recovery to troubleshoot ambiguous assemblies). These three sequencing runs were all conducted for 72 h with flow cells harboring > 1400 pores at run start. The resulting datasets were named hereafter PCR, TAG, and LIG libraries, according to library preparation strategies (i, ii and iii above). Basecalling (i.e. translating the nanopore electronic raw signal of the sequencer into nucleotide bases) of the *fast5* files was conducted with Guppy v6.1.1 (Oxford Nanopore Technologies) set with the « super accuracy» mode (i.e. the highest accuracy model *–config dna_r9.4.1_450bps_sup.cfg*) and demultiplexing conducted by assessing barcodes on the front and rear of the reads. For simplicity and to examine read properties from different library strategies (see further below), no subsequent read filtering was applied. All reads, classified by default as ‘fail’ or ‘pass’ by Guppy based on mean Qscore threshold of 7, were merged into a common *fastQ* file for all analyses (see Additional file 2 for further details on fail vs. pass reads output and quality). We chose to keep fail reads to maximize coverage and read length available for genome circularisation, as well as to avoid further ramification of read categories to analyse separately (see Additional file 2 for fail read mappability and quality).

### Illumina sequencing

DNA libraries were produced via tagmentation with a Nextera® XT kit and sequenced in 2 × 150 bp on a NextSeq system (Illumina, San Diego, CA) at Microsynth (Balgach, Switzerland). Average base and sequence output per strain was < 1 Gbp for > 7 M PE reads (i.e. > 3.5 M pairs of Forward and Reverse reads), respectively.

One strain (CD3406) was sequenced in 2 × 250 bp on a Hiseq system (Illumina, San diego, CA) at Genoscreen (Lille, France) with a lower output of < 0.2 Gbp and < 0.7 M PE reads (earlier study of [24]). DNA extraction for Illumina sequencing followed the same protocol as for nanopore sequencing (further above) but from smaller culture volumes.

### Library assemblies

Demultiplexed *fastQ* files from the PCR and TAG libraries for each of 12 bacterial strains were assembled per library with (1) Unicycler for Illumina short reads-only within the CELIA pipeline [25], (2) Unicycler for hybrid Illumina short-reads followed by scaffolding with long nanopore reads and (3) Flye and MetaFlye for nanopore long reads-only. Unicycler v0.4.8 [7] was used because it includes a polishing step with Pilon [26] (the subsequent version v0.5.0 does not include Illumina polishing) and ran with *–mode conservative*. Flye v2.9 [27, 28] was used with the option *–nano-raw*. MetaFlye was run by further adding the *–meta* flag for assembly in uneven read coverage mode. Only the best results from Flye/MetaFlye were reported (Table 2, see Additional file 3 Table S2 for further details on Flye/MetaFlye output differences). To do so, assemblies (Illumina vs. hybrid vs. nanopore) were scored for circular molecules by checking Unicycler’s « unicycler.log» file (molecules indicated as « complete», which are also flagged as « circular = true» within the *fastA* titles of the assembled contig file) and Flye/MetaFlye’s « assembly_info.txt» file (circular molecule indicated as yes « Y»). When molecules were incomplete (i.e. not circular), BLASTn [29] was used to identify the longest assembled contig in individual assembly files. Contigs were compared to the curated genomes (see below) to document any missassemblies and/or observed genomic variation (Table 2, see superscript indices).

### Replicon assessment

Complete circular replicons (chromosomes or plasmids) for a given strain and across libraries and/or assemblers were rotated from a common single-copy gene or non-repeated intergenic region with perl script fasta_shift.pl from the FASTA-tools package [30]. Rotated molecules were then aligned in a pairwise manner via BLASTn or Minimap2 to produce alignment reports and dotplots [31], which were examined for the presence of breaks revealing assembly differences. Large breaks, i.e. those comprising large physical distances (i.e. nucleotide stretch), were investigated via mapping with BWA-MEM and the resulting *bam* file visualized in IGV [32]. Smaller breaks were verified for tandem repeat variation with TRF [9] (command line as in next section) or for homology against raw reads (or Genbank’s nr) after extraction of the corresponding genomic region with fasta_sub.pl from the FASTA-tools package [30]. BLASTn results were also downloaded as *xml* file for visualization of eventual genomic segment(s) rearrangement(s) or presence of large interspersed repeats (Kbp repeated segments) with Kablammo [33]. A summary table (Additional file 3 Table S5) was also prepared to report overall genome complexity for each strain by documenting the number of 16S copies per chromosome, the number of long repeated segments per chromosome (> 1,000 bp in BLASTn of the chromosomes on themselves), detected tandem repeats using TRF and number of plasmids. Insertion sequences, prophages and CRISPR (clustered regularly interspaced short palindromic repeats), which represent additional small repetitive regions that may occur in bacterial genomes and may participate to genome complexity (i.e. horizontal transfer, duplication, recombination), were documented with the ISfinder database by retaining detected regions with evalue < 0.0001 [34], PHASTER [35] and CRISPR-Cas +  + [36], respectively.

### Curated genomes

When Unicycler and Flye/MetaFlye assemblies agreed, we picked results from the former as best replicon for simplicity because its contigs are already polished (Table 2, Additional file 3 Table S4). When Flye/MetaFlye provided a better assembly than Unicycler, polishing was first conducted with nanopore reads with Medaka [37] using the model file command *-m r941_min_sup_g507*, then followed by Illumina polishing with Polypolish [38]. For the latter, Illumina reads were mapped to the medaka-polished contig with BWA-MEM [39] to generate *sam* files. Achieved nanopore coverage for validated circular chromosomes had a minimum nanopore coverage of 42X, although most greatly exceeded this value (see Additional file 3 Table S3; not shown in Table 2 for clarity). Likewise, Illumina coverage was at a minimum of 62X, but greatly exceeded this value in most strains (not shown). For two strains whose chromosome was near complete but failed to circularize, even when the PCR and TAG libraries were combined (i.e. SAF3325, SF1671), we also sorted reads from the pooled LIG run (i.e. reassembly with LIG-only data, or combined with TAG and PCR) (Additional file 3 Table S3). To do so, preliminary assemblies were mapped with Minimap v2-2.24 (option ‘*-ax map-ont*’, [40]) and the resulting *sam* file filtered with msamtools [41] for reads > 5,000 bp mapping at > 80% cover and > 95% identity (*filter -S -b -l 5000 -p 95 -z 80*). Filtered, mapped reads output in *bam* format, were then extracted into *fastQ* files with samtools v1.9 [42] (see commands further below) for reassembly with Flye/MetaFlye. In a few instances, and for added confidence (i.e. seeking congruence), assemblies were also produced with wtdbg2 [6] and canu [5] (not shown). Finally, following mapping analysis on all curated genomes (see below), unmapped reads were segregated in a *fast*Q file and assembled with Flye/MetaFlye to verify if any replicons may have been overlooked.

### Read attributes

Reads were mapped to the established curated genomes using Minimap2. The resulting *sam/bam* mapping files were then post-processed with samtools to extract mapped vs. unmapped reads using functions *view* and *fastq* (with flags *-F 4* or *-f 4*, respectively). The mapping files were also processed with msamtools with the *summary* function to obtain reads’ taxonomic identity (i.e. which genome a read mapped to). This allowed for estimation of sample-to-read misassignment (i.e. barcode leakage) by comparing read’s mapped taxonomy to its barcode identity reported by Guppy. Tandem repeats were also computed for each of the read of the three libraries (PCR, TAG, and LIG) with TRF v.4.09.1 set with a maximum 2000 bp pattern detection (option ‘*2 5 7 80 10 50 2000 -d -h’*, [9]). The TRF report was then parsed to join overlapping tandems and compute total tandem length per read as well as their location with a custom script written in R [43] named TROP (Tandem Repeat Overlap Parser [10]). All of the above information, a read mapped vs unmapped status, its mapped taxonomy and tandem content, was then merged via read labels to Guppy’s « *sequencing_summary.txt*» file in order to create a synthesis file (available at [44]) from which to access all associated read attributes (e.g. read’s length, mean Qscore, barcode identity) and compute different summary statistics (Tables 3, 4, 5 and 6, Figs. 2, 3 and 4, Additional file 2, 3 and 4). A second synthesis file includes the location of the non-overlapping tandems along the reads (data displayed in Fig. 5 and available at [44]).

### Computing

Data analyses, assemblies and polishing tools were run on Ifremer’s supercomputer DATARMOR located within the institute’s bioinformatics core facility called Sebimer. Basecalling of nanopore’s raw signal with Guppy was conducted on an NVIDIA Tesla V100 (PCIe 32GB) GPU Accelerator. Plots and tables were produced in R with package ggplot2 [45] and data.table [46].

### Supplementary Information

**Additional file 1. **Preparation kits.

**Additional file 2. **Fail vs. Pass reads.

**Additional file 3. **Assembly details and chromosome attributes.

**Additional file 4. **Barcoding statistics.

**Additional file 5. **DNA extraction protocol.

## Data Availability

Raw (*fastQ*) nanopore and Illumina data are available under ENA’s Bioproject PRJEB60605. Raw (*fast5*) nanopore signal data, curated genomes and syntheses files with read properties are available on Ifremer’s repository [44]. The curated chromosome and plasmids of strain *Serratia proteomaculens* (CD3406, previously published [24]) were updated under NCBI’s Bioproject PRJNA686006 and available under Genbank's accessions CP117168-CP117175.

## Abbreviations

Gbp: Gigabase pair
IGV: Integrated Genome Viewer
Kbp: Kilobase pair
LIG: Ligation-based library
Mbp: Million base pair
ONT: Oxford Nanopore Technology
PCR: PCR Amplification-based library
PE: Illumina paired-ends
SV: Structural Variation
TAG: Tagmentation-based library
