# Supplementary material for: A comparison of Oxford nanopore library strategies for bacterial genomics
Source: BMC Genomics. 2023 Oct 20;24:627. doi: 10.1186/s12864-023-09729-z (PMC10589936; doi:10.1186/s12864-023-09729-z)
Supplement: Supplementary file 1 — Additional file 1. Preparation kits. [file 12864_2023_9729_MOESM1_ESM.pdf]

Additional File 1 : Preparation kits and DNA extractions

Table S1. Kit summary

Kit name	Ref	DNA	Time[1]	Barcode Adapter	Sequencing Adapter	Consumables	Study name
"Ligation Sequencing"<br>+ "Native barcoding[2,3]"	SQK-LSK110<br>NA[2,3]	1000 ng	140 min	NA[2,3]	AMII	FFPE, ER/dA<br>LIG1, LIG2	LIG
"Ligation Sequencing"<br>+ "PCR barcoding"	SQK-LSK110<br>+ EXP-PBC001	1000 ng	85 min<br>+ PCR	BCA + BC01-BC12	AMX-F	FFPE, ER/dA[4]<br>LIG1, LIG2, LR-Taq	PCR
"Rapid Barcoding"<br>(="Native tagmentation")	SQK-RBK004	400 ng	10 min	RB01-RB12	RAP	--	TAG

Kits available to produce up to 6 libraries of 12 samples using ligation, tagmention or amplification at the time of the study. Magnetic beads are required for all kits and thus are not listed in consumables. The library names used in the present study are given in the last column as LIG, TAG or PCR (see manuscript introduction).

[1]ONT-indicated minimum time
[2]Native Barcoding expansion NOT AVAILABLE for SQK-LSK110. The required expansion for the previous kit SQK-LSK109 was named EXP-NBD104 and contained adapters NBD01-NBD12.
[3]A pooled library without barcodes was prepared via ligation (SQK-LSK110) and sorted bioinformatically (see methods for further information)
[4]End-repair is conducted twice (prior to PCR adapter ligation and prior to sequencing adapter ligation)

Abbreviations :

AMII = Adapeter Mix II
AMX-F = Adapter Mix F
BC = Barcode
BCA = Barcode Adapter
ER/dA = NEBNext Ultra II End Repair / dA-Tailing Module
FFPE = NEBNext FFPE DNA Repair Mix
LIG1 = NEB Blunt/TA DNA Ligase
LIG2 = NEBNext Quick T4 DNA ligase
LR-Taq = Long Range Taq
RAP = Rapid Sequencing Adapter Attachment
RB = Rapid Barcode
