# Supplementary material for: A comparison of Oxford nanopore library strategies for bacterial genomics
Source: BMC Genomics. 2023 Oct 20;24:627. doi: 10.1186/s12864-023-09729-z (PMC10589936; doi:10.1186/s12864-023-09729-z)

Additional File 2 : Fail vs. Pass reads

Table S2. Run output statistics.

Library	Scored	Class	Read count	%	Cum. Length (bp)	%
LIG	FAIL	Mapped	275,904	4.2 %	1,613,454,327	4.6 %
		Unmapped	454,443	7.0 %	1,514,268,135	4.3 %
	PASS	Mapped	5,780,413	88.7 %	32,011,136,267	91.1 %
		Unmapped	5,107	0.1 %	4,543,242	0.0 %
PCR	FAIL	Mapped	30,439	0.4 %	96,302,737	1.3 %
		Unmapped	567,959	8.2 %	520,530,625	7.2 %
	PASS	Mapped	1,541,232	22.3 %	4,697,920,761	64.7 %
		Unmapped	4,776,264	69.1 %	1,951,782,865	26.9 %
TAG	FAIL	Mapped	100,891	4.7 %	691,839,241	5.6 %
		Unmapped	195,493	9.1 %	642,987,891	5.2 %
	PASS	Mapped	1,774,532	82.6 %	11,030,107,949	88.9 %
		Unmapped	77,791	3.6 %	39,792,911	0.3 %

Read counts and cumulative read length (bp) for the three flow cell runs with library preparation strategy LIG, PCR and TAG.

Figure S1. Mosaic plot. Visualization of read count abundance for mapped (blue) vs. unmapped (red) for pass vs. fail read categories per library strategy. The mosaic plot was prepared with the R package vcd (Meyer et al. 2023). Note the important proportion of unmapped reads in the PCR library. Note as well the large number of reads in libraries LIG and PCR vs. TAG (i.e. seen as the width) and presence of mapped (i.e. biological) reads within fail reads. Reads were classified as pass or fail by Guppy and determined as mapped or unmapped by Minimap2 based on mapping to curated genomes.

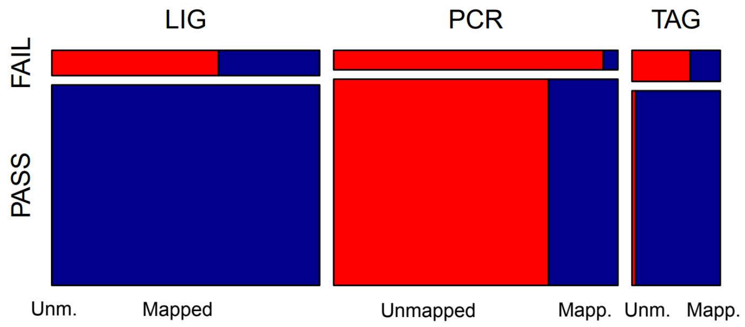

Meyer D, Zeileis A, Hornik K. vcd : Visualizing Categorical Data. R package version 1.4-11. 2023 ; https://CRAN.R-project.org/package=vcd.

Figure S2. Read quality fail vs. pass. (a) Read mean Qscore obtained from Guppy's basecalling summary file, (b) read match percent identity based on mapping to curated genome with Minimap2, (c) read length in Kbp and (d) within read tandem length in Kbp. Note that while fail reads are generally lower in quality, several do overlap in high range values (see the long, skinny upper tails). Fail and pass reads exhibit similar read length distribution and both contain reads with very long tandems (long upper tails in d).

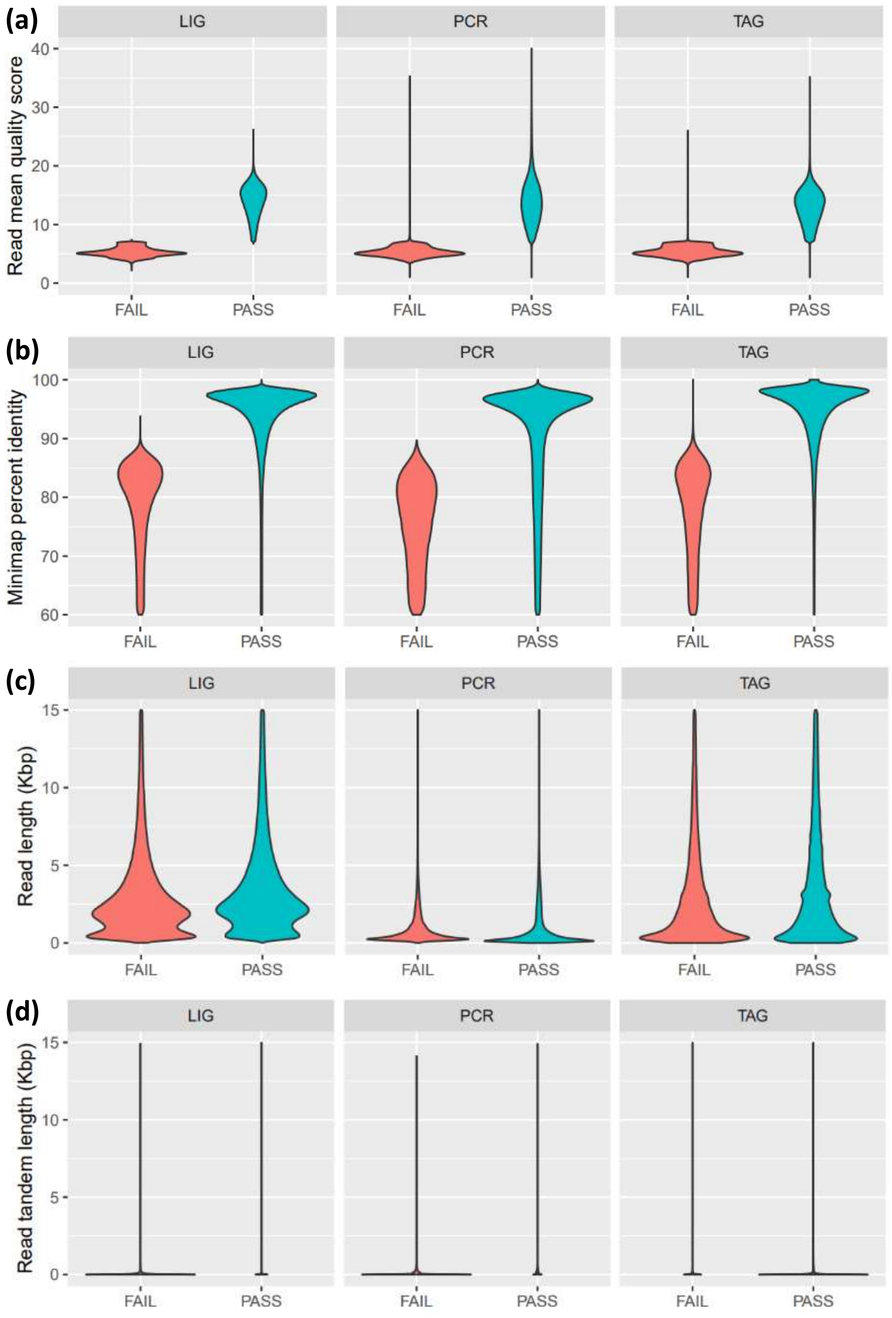

Supplement: Supplementary file 2 — Additional file 2. Fail vs. Pass reads. [file 12864_2023_9729_MOESM2_ESM.pdf]
