# Supplementary material for: A comparison of Oxford nanopore library strategies for bacterial genomics
Source: BMC Genomics. 2023 Oct 20;24:627. doi: 10.1186/s12864-023-09729-z (PMC10589936; doi:10.1186/s12864-023-09729-z)
Supplement: Supplementary file 3 — Additional file 3. Assembly details and chromosome attributes. [file 12864_2023_9729_MOESM3_ESM.pdf]

Additional File 3 : Assembly details and chromosome attributes

Table S3 : Flye vs. MetaFlye library assemblies

		SF2022	SF1671	SF1039	SAF3333	SAF3325	MIP2602	MIP2473	MIP2461	MIP2422	HIS2824	EBP3064	CD3406
PCR	Largest Frag (bp)	**2,157,934**	**1,940,706**	**21,540**	**1,384,924**	2,404,472	2,466,113	**1,999,148**	**57,456**	**190,828**	**2,966,192**	**50,742**	**1,461,548**
(Flye)	Circular?	N	N	N	N	N	N	N	N	N	N	N	N
	Cov. Largest Frag	51	27	3	116	125	64	128	3	10	82	10	11
PCR	Largest Frag (bp)	2,157,932	1,934,734	21,567	1,384,926	**2,408,904**	**3,556,347**	1,998,973	57,487	248,880	2,966,186	50,742	1,461,574
(MetaFlye)	Circular?	N	N	N	N	N	N	N	N	N	N	N	N
	Cov. Largest Frag	51	27	3	116	124	65	127	3	10	82	10	11
TAG	Largest Frag (bp)	**3,408,591**	**497,793**	**5,043,970**	**2,313,803**	4,177,807	**6,337,130**	--	**4,813,116**	**3,342,645**	**4,187,919**	**5,406,564**	**5,677,442**
(Flye)	Circular?	Y	N	Y	Y	N	Y	--	Y	Y	Y	Y	Y
	Cov. Largest Frag	20	4	57	95	46	153	--	94	32	385	58	119
TAG	Largest Frag (bp)	3,408,589	497,795	5,043,981	1,392,773	**4,196,714***	6,337,153	**3,241,428**	4,813,118	3,342,660	4,187,917	5,406,601	5,677,403
(MetaFlye)	Circular?	Y	N	Y	N	Y	Y	Y	Y	Y	Y	Y	Y
	Cov. Largest Frag	20	4	57	89	46	152	1,007	94	32	385	60	119
PCR+TAG	Largest Frag (bp)	3,409,220	6,441,768	5,043,986	2,313,842	4,196,595	6,337,341	--	4,813,038	3,342,371	4,188,666	5,406,605	5,677,451
(Flye)	Circular?	Y	Y	Y	Y	Y	Y	--	Y	Y	Y	Y	Y
	Cov. Largest Frag	72	36	59	222	195	218	--	95	42	477	65	134
PCR+TAG	Largest Frag (bp)	3,409,281	6,441,765	5,043,985	2,313,841	4,196,595	6,337,330	--	4,813,032	3,342,370	4,188,666	5,406,606	5,677,468
(MetaFlye)	Circular?	Y	Y	Y	Y	Y	Y	--	Y	Y	Y	Y	Y
	Cov. Largest Frag	74	36	59	222	195	218	--	95	42	477	65	134
LIG	Largest Frag (bp)	NA	**6,451,109***	NA	NA	--	NA	NA	NA	NA	NA	NA	NA
(Flye)	Circular?	NA	Y	NA	NA	--	NA	NA	NA	NA	NA	NA	NA
	Cov. Largest Frag	NA	249	NA	NA	--	NA	NA	NA	NA	NA	NA	NA
LIG	Largest Frag (bp)	NA	6,451,104	NA	NA	4,157,835	NA	NA	NA	NA	NA	NA	NA
(MetaFlye)	Circular?	NA	Y	NA	NA	Y	NA	NA	NA	NA	NA	NA	NA
	Cov. Largest Frag	NA	249	NA	NA	799	NA	NA	NA	NA	NA	NA	NA

Results of Flye and MetaFlye assemblies with default parameters for individual and/or combination of libraries. Curated replicons reported in Table 2 and Table S4 are highlighted in bold. For LIG, reads were sorted via mapping for SF1671 and SAF3325 for reassembly (see methods) since PCR and TAG libraries failed to circularize the main chromosome when run individually (« N » for No). Note that Flye crashed for MIP2473 with TAG or PCR+TAG with the error "No disjointigs were assembled", which is possibly due to variable or high coverage issues (github.com/fenderglass/Flye/issues/128). The same error occured for SAF3325 with LIG or LIG+TAG+PCR. Note that MIP2473 circularized with MetaFlye for the TAG library, but led to anormally high coverage of 1007X and also crashed with TAG+PCR (same error as with Flye, see above). Note that SAF3325 with LIG or LIG+TAG+PCR crashed with Flye but not MetaFlye. LIG reads were not sorted for the remainder of samples since it was not needed (NA not attempted). Finally, note that LIG assembly of SF1671 led to a chromosome 10 Kbp longer than with PCR+TAG. Note that LIG assembly of SAF3325 with LIG led to a 40 Kbp shorter chromosome. Based on mapping analysis and IGV visualization for these two strains, we retained genomes flagged with an asterisk (*) for further polishing.

Table S4 : Assemblies of barcoded libraries (longest or circular fragment bp)

Strain	Replicon	PE	PE+PCR	PE+TAG	PCR	TAG	Curated
Serratia proteamaculans (CD3406)	Chrom.	376,018	376,018	○ 5,678,535*	1,461,548	○ 5,677,442	○ 5,678,535
	Plasm.1	109,734	○ 110,418	○ 110,418*	○ 110,397	○ 110,368	○ 110,418
	Plasm.2	50,925	○ 51,609	○ 51,609*	○ 51,603	○ 51,584	○ 51,609
	Plasm.3	○ 46,588	○ 46,588	○ 46,588*	○ 46,580	○ 46,558	○ 46,588
	Plasm.4	○ 23,984	○ 23,984	○ 23,984*	○ 23,970	○ 47,926[R]	○ 23,984
	Plasm.5	7,443	7,443	○ 8,500*	4,039	○ 8,487	○ 8,500
	Plasm.6	○ 7,000	○ 7,000	○ 7,000*	9,985[M]	3,449	○ 7,000
	Plasm.7	○ 3,223	○ 3,223	○ 3,223*	○ 3,223	○ 3,218	○ 3,223
Serratia proteamaculans (EBP3064)	Chrom.	868,704	1,154,321	○ 5,376,166[M]	50,742	○ 5,406,564*	○ 5,407,376
	Plasm.1	○ 108,855	○ 108,855	○ 108,855*	71,537	○ 108,805	○ 108,855
	Plasm.2	○ 5,746	○ 5,746	○ 5,746*	○ 11,488[R]	○ 5,738	○ 5,746
	Plasm.3	○ 3,223	○ 3,223	○ 3,223*	○ 3,126	○ 3,219	○ 3,223
Serratia fonticola (MIP2602)	Chrom.	449,831	5,586,596[T]	○ 6,338,624*	3,556,347	○ 6,337,130	○ 6,338,624
	Plasm.	○ 25,589	○ 25,589	○ 25,589*	○ 25,586	○ 51,165[R]	○ 25,589
Morganella morganii (HIS2824)	Chrom.	736,371	2,951,888[T]	○ 4,190,719[T]	2,966,192[T]	○ 4,187,917[T]*	○ 4,190,812
	Plasm.	○ 3,126	○ 3,126	○ 3,126*	○ 3,123[M]	○ 3,122	○ 3,126

Hafnia paralvei (MIP2461)	Chrom.	1,129,120[T]	1,529,248	◯ 4,814,021*	57,456	◯ 4,813,116[A,M]	◯ 4,814,021
	Plasm.1	42,458	108,563[T,M]	◯ 110,923*	14,016[M]	◯ 110,903	◯ 110,923
	Plasm.2	39,016	39,016[N,M]	◯ 39,765*	2,592	◯ 39,902	◯ 39,765
	Plasm.3	◯ 6,176	◯ 6,176	◯ 6,176*	◯ 6,171	◯ 6,172	◯ 6,176
Photobact. phosphoreum (MIP2473)	Chrom.1	405,078	2,455,384[T]	2,926,596[M]	1,999,148	◯ 3,241,428[T]*	◯ 3,242,459
	Chrom.2	292,046	◯ 1,420,734[T]	◯ 1,421,225*	◯ 1,416,489[M]	◯ 1,420,800	◯ 1,421,225
Shewanella baltica (SF1039)	Chrom.	236,238	310,670	◯ 5,044,058[T]*	21,567	◯ 5,043,970[T]	◯ 5,044,058
	Plasm.	138,932[N]	138,932[N]	◯ 141,179*	9,173	◯ 141,142	◯ 141,179
Pseudomonas fluorescens (SF1671)	Chrom.	285,740	2,470,621[M]	1,162,926[M]	1,940,706	497,793	◯ 6,452,312
Weeksellaceae (MIP2422)	Chrom.	328,898	942,011	◯ 3,342,924*	190,828	◯ 3,342,645	◯ 3,342,924
Bacillus velezensis (SAF3325)	Chrom.	86,1947	1,297,870	◯ 4,197,796*	2,408,904[T,M]	◯ 4,196,714[T,M]	◯ 4,197,796
Lactococcus piscium (SAF3333)	Chrom.	283,355	1,369,996	2,305,486[X,N]	1,384,924	◯ 2,313,842*	◯ 2,314,465
	Plasm.	8,007	◯ 11,916	◯ 11,916*	◯ 11,908	◯ 23,823[R]	◯ 11,916
Carnobact. maltaromaticum (SF2022)	Chrom.	711,623	3,402,107	◯ 3,410,292*	2,157,934[T]	◯ 3,408,591	◯ 3,410,292
	Plasm.	92,598[N]	◯ 92,712	◯ 92,683*	◯ 92,680	◯ 92,645	◯ 92,683

Longest fragment obtained for Illumina Paired-Ends (PE), hybrid and nanopore assemblies with Unicycler (PE, PE+PCR, PE+TAG) and Flye/MetaFlye (PCR and TAG) for the barcoded libraries (no barcoding was possible for LIG, see methods). Assemblies are reported as Incomplete molecule (no symbol) or Complete circular molecule (◯). Genomes kept as curated replicons following assembly assessment and polishing (see methods) are flagged with an asterisk (*). Assembly notes are documented with the following indices : Near circular molecule (N), Presence of small missassemblies (M), Tandem repeat variation (T), Large chromosome inversion (X), Plasmid concatemer (R), Presence of artifactual tandems (A). Note that SF1671 and SAF3325 were reassembled with sorted LIG data for circularization and/or further confirmation of the chromosome scaffold. A concise version of this table is found within the manuscript as Table 2. Strain, replicon and curated size are highlighted in grey for greater clarity.

Table S5. Chromosome attributes

	Chrom. length	GC%	16S rDNA	HSPs	Plasmid	Prophage	CRISPR	IS	IS/Mbp	TR	TR(total bp)	TR(%)
CD3406	5,678,535 bp	55.08	7	354	**7**	3	0	96	16.9	140	5,071 bp	0.1
EBP3064	5,376,166 bp	55.21	7	208	3	1	0	51	9.5	132	4,017 bp	0.1
MIP2602	6,338,624 bp	53.54	7	**1246**	1	**8**	0	178	28.1	208	12,013 bp	0.2
HIS2824	4,190,812 bp	50.35	7	172	1	3	0	25	6.0	256	14,040 bp	0.3
MIP2461	4,814,021 bp	47.96	8	160	3	3	0	81	16.8	180	10,537 bp	0.2
MIP2473_chr1	3,242,459 bp	40.54	**24**	894	0	0	0	23	7.1	267	**19,777 bp**	0.6
MIP2473_chr2	1,421,225 bp	37.56	0	122	0	0	**2**	24	16.9	165	10,992 bp	**0.8**
SF1039	5,044,058 bp	46.38	10	602	1	0	1	106	21.0	**564**	17,032 bp	0.3
SF1671	6,452,312 bp	**59.91**	6	344	0	1	0	**192**	**29.8**	405	16,635 bp	0.3
MIP2422	3,342,924 bp	36.03	7	256	0	0	**2**	2	0.6	454	17,091 bp	0.5
SAF3325	4,197,796 bp	46.98	9	200	0	3	0	4	1.0	130	3,485 bp	0.1
SAF3333	2,314,465 bp	38.48	4	243	1	0	0	40	17.3	91	6,236 bp	0.3
SF2022	3,410,292 bp	34.48	6	102	1	0	0	2	0.6	260	17,180 bp	0.5

Summary of chromosome attributes representing potential indicators of genomic complexity. These include GC%, the number of 16S rDNA copies, BLASTn secondary high scoring pairs (HSPs) >1,000 bp, number of plasmids, Prophage, Insertion sequences (IS), Clustered Regularly Interspaced Short Palindromic Repeats (CRISPRs) and tandem repeats (TR) (number, cumulative base pair and percentage of chromosome). Chromosomes that could not correctly be assembled with hybrid PE+TAG data are shaded in grey (i.e. corresponding to chromosome that could only be correctly assembled with nanopore data only with Flye/MetaFlye). Note that while some attributes of the difficult genomes (those highlighted in grey) show some large values, several genomes that assembled without issue in hybrid approach also displayed large values. Note that GC% values above are derived from curated chromosome and may slightly differ from GC% values reported in Table 1 from preliminary Illumina assemblies.
