# Supplementary material for: A comparison of Oxford nanopore library strategies for bacterial genomics
Source: BMC Genomics. 2023 Oct 20;24:627. doi: 10.1186/s12864-023-09729-z (PMC10589936; doi:10.1186/s12864-023-09729-z)
Supplement: Supplementary file 4 — Additional file 4. Barcoding statistics. [file 12864_2023_9729_MOESM4_ESM.pdf]

Additional File 4 : Barcoding statistics

Table S6. Flow cell output per barcode and library strategy

	Barcode	Strain	Read count	Proportion	Cum. Length (bp)	Proportion
LIG	Unclassified	NA	6,515,867	100%	35,143,401,971	100%
PCR	Barcode 01	SF2022	334,500	4.8 %	297,924,024	4.1 %
PCR	Barcode 02	EBP3064	535,424	7.7 %	200,516,310	2.8 %
PCR	Barcode 03	MIP2461	871,706	12.6 %	372,537,180	5.1 %
PCR	Barcode 04	CD3406	946,118	13.7 %	479,602,850	6.6 %
PCR	Barcode 05	SAF3325	381,442	5.5 %	731,441,263	10.1 %
PCR	Barcode 06	SF1671	258,028	3.7 %	299,706,317	4.1 %
PCR	Barcode 07	MIP2602	206,087	3.0 %	500,645,873	6.9 %
PCR	Barcode 08	HIS2824	322,032	4.7 %	472,899,079	6.5 %
PCR	Barcode 09	SF1039	369,108	5.3 %	147,782,494	2.0 %
PCR	Barcode 10	MIP2422	703,255	10.2 %	322,704,699	4.4 %
PCR	Barcode 11	MIP2473	354,772	5.1 %	721,654,099	9.9 %
PCR	Barcode 12	SAF3333	188,771	2.7 %	359,455,753	4.9 %
PCR	Unclassified	NA	1,444,651	20.9 %	2,359,667,047	32.5 %
TAG	Barcode 01	SF2022	17,452	0.8 %	74,385,641	0.6 %
TAG	Barcode 02	EBP3064	135,176	6.3 %	374,833,014	3.0 %
TAG	Barcode 03	MIP2461	53,260	2.5 %	523,144,075	4.2 %
TAG	Barcode 04	CD3406	104,253	4.9 %	938,433,752	7.6 %
TAG	Barcode 05	SAF3325	112,356	5.2 %	208,163,263	1.7 %
TAG	Barcode 06	SF1671	4,781	0.2 %	23,543,715	0.2 %
TAG	Barcode 07	MIP2602	154,591	7.2 %	1,009,017,033	8.1 %
TAG	Barcode 08	HIS2824	354,087	16.5 %	1,745,005,781	14.1 %
TAG	Barcode 09	SF1039	114,489	5.3 %	315,866,763	2.5 %
TAG	Barcode 10	MIP2422	16,312	0.8 %	113,024,688	0.9 %
TAG	Barcode 11	MIP2473	528,929	24.6 %	4,485,515,848	36.2 %
TAG	Barcode 12	SAF3333	100,598	4.7 %	257,901,404	2.1 %
TAG	Unclassified	NA	452,423	21.1 %	2,335,893,015	18.8 %

Number of reads in millions (M) and cumulative read length (bp) per barcode/strain for library runs LIG, PCR and TAG. The category 'Unclassified' corresponds to reads for which Guppy could not successfully call a barcode. Note that barcoding was not possible for LIG (see methods).

Table S7. Classified reads breakdown per barcode and run following mapping.

	Barcode	Strain	Correct	Leaked	Unmapped
LIG	NA	NA	NA	NA	NA
PCR	Barcode 01	SF2022	17.7 %	1.6 %	80.7 %
	Barcode 02	EBP3064	1.9 %	1.6 %	96.5 %
	Barcode 03	MIP2461	0.4 %	1.4 %	98.1 %
	Barcode 04	CD3406	2.4 %	1.4 %	96.2 %
	Barcode 05	SAF3325	72.1 %	0.7 %	27.2 %
	Barcode 06	SF1671	19.9 %	1.2 %	78.9 %
	Barcode 07	MIP2602	63.2 %	1.2 %	35.6 %
	Barcode 08	HIS2824	42.0 %	0.9 %	57.1 %
	Barcode 09	SF1039	2.0 %	1.4 %	96.7 %
	Barcode 10	MIP2422	1.5 %	1.4 %	97.2 %
	Barcode 11	MIP2473	96.8 %	0.7 %	2.5 %
	Barcode 12	SAF3333	45.3 %	0.9 %	53.8 %
	Mean ± sd		30.4 % ± 33.0	1.2 % ± 0.3	68.4 % ± 32.7
TAG	Barcode 01	SF2022	73.3 %	0.6 %	26.2 %
	Barcode 02	EBP3064	90.3 %	4.8 %	5.0 %
	Barcode 03	MIP2461	93.6 %	0.6 %	5.8 %
	Barcode 04	CD3406	89.1 %	5.8 %	5.2 %
	Barcode 05	SAF3325	95.8 %	0.5 %	3.7 %
	Barcode 06	SF1671	43.4 %	2.7 %	53.9 %
	Barcode 07	MIP2602	89.0 %	0.8 %	10.2 %
	Barcode 08	HIS2824	98.0 %	0.1 %	1.9 %
	Barcode 09	SF1039	94.3 %	0.2 %	5.5 %
	Barcode 10	MIP2422	92.6 %	0.4 %	7.0 %
	Barcode 11	MIP2473	97.8 %	0.1 %	2.1 %
	Barcode 12	SAF3333	92.6 %	4.8 %	2.5 %
	Mean ± sd		87.5 % ± 15.3	1.8 % ± 2.1	10.7 % ± 15.1

Proportion of correctly classified (‘correct’ column) and incorrectly classified (‘leakage’ column) reads based on mapping to curated genomes with Minimap2. Reads for which a barcode was detected by Guppy but did not match any of the curated genomes represent artifactual or very low quality reads (‘Unmapped’ column). Percentage is calculated based on read number.

Table S8. Classified read lengths per read category

		Correct	Leaked	Unmapped
PCR	mean (match)	2,788 bp (2,558 bp)	3,793 bp (2,531 bp)	347 bp (0 bp)
	max (match)	64,795 bp (22,193 bp)	85,916 bp (18,253 bp)	299,864 bp (0 bp)
TAG	mean (match)	6,218 bp (5,948 bp)	3,376 bp (2,861 bp)	221 bp (0 bp)
	max (match)	347,121 bp (127,754 bp)	80,068 bp (78,898 bp)	205,465 bp (0 bp)

Classified read lengths computed as mean and max based on basecalled length (i.e. the entire read) and the actual nucleotide length successfully mapping to curated genome (match in parenthesis) computed by Minimap2. Lengths are reported in base pair. Note that unmapped reads (artifactual reads or very low quality reads) are in average very short but some can be very long. Note as well that leaked reads show a wide range of sizes, thus no size bias in Guppy's barcode classification seem to happen. See Table S7 caption for further details on categories 'Correct', 'Leaked' and 'Unmapped'.

Table S9. Reads lengths (bp) per barcode : Basecalled vs. Mapped

			Guppy		Minimap	
	Barcode	Strain	mean length ± sd	max length (match)	mean length ± sd	max length (match)
PCR	Barcode01	SF2022	891 ± 1,621	299,864 (21,144)	3,741 ± 2,009	30,483 (21,144)
	Barcode02	EBP3064	375 ± 780	203,045 (14,950)	3,162 ± 1,608	38,947 (11,449)
	Barcode03	MIP2461	427 ± 641	84,729 (17,794)	3,756 ± 2,675	33,404 (27,226)
	Barcode04	CD3406	507 ± 851	25,164 (19,349)	4,147 ± 2,333	35,135 (19,986)
	Barcode05	SAF3325	1,918 ± 1,445	34,080 (15,166)	2,716 ± 1,321	21,097 (12,652)
	Barcode06	SF1671	1,162 ± 1,924	65,089 (22,193)	4,705 ± 2,255	38,331 (22,193)
	Barcode07	MIP2602	2,429 ± 2,021	24,185 (19,598)	3,794 ± 1,868	34,403 (19,598)
	Barcode08	HIS2824	1,468 ± 1,574	64,795 (18,308)	3,139 ± 1,517	84,729 (18,308)
	Barcode09	SF1039	400 ± 648	85,916 (17,656)	1,978 ± 1,787	24,986 (17,656)
	Barcode10	MIP2422	459 ± 754	113,396 (18,407)	3,749 ± 5,215	345,053 (18,407)
	Barcode11	MIP2473	2,034 ± 1,314	35,938 (20,444)	2,256 ± 1,650	488,457 (20,444)
	Barcode12	SAF3333	1,904 ± 2,218	35,613 (20,213)	4,068 ± 2,157	35,613 (20,213)
TAG	Barcode01	SF2022	4,262 ± 7,346	78,826 (69,146)	5,494 ± 7,873	80,068 (78,898)
	Barcode02	EBP3064	2,773 ± 3,116	166,218 (44,561)	3,004 ± 3,081	94,446 (39,894)
	Barcode03	MIP2461	9,822 ± 11,998	126,310 (125,478)	10,427 ± 12,070	126,310 (125,478)
	Barcode04	CD3406	9,002 ± 11,262	128,974 (127,754)	9,440 ± 11,407	128,974 (127,754)
	Barcode05	SAF3325	1,853 ± 2,547	78,436 (67,338)	1,954 ± 2,560	69,608 (45,719)
	Barcode06	SF1671	4,924 ± 10,181	80,349 (78,842)	11,222 ± 13,207	80,349 (78,842)
	Barcode07	MIP2602	6,527 ± 8,594	205,465 (114,683)	7,362 ± 8,817	199,547 (114,683)
	Barcode08	HIS2824	4,928 ± 5,795	347,121 (86,850)	5,098 ± 5,844	34,7121 (86,850)
	Barcode09	SF1039	2,759 ± 4,423	76,398 (66,696)	2,998 ± 4,550	76,398 (66,696)
	Barcode10	MIP2422	6,929 ± 8,172	95,982 (95,343)	7,911 ± 10,971	301,849 (95,343)
	Barcode11	MIP2473	8,480 ± 9,983	159,582 (122,355)	8,750 ± 10,079	394,082 (122,355)
	Barcode12	SAF3333	2,564 ± 4,595	80,068 (78,898)	2,630 ± 4,526	98,136 (68,890)
LIG	Barcode01	SF2022	NA	NA	10,208 ± 12,343	206,449 (118,432)
	Barcode02	EBP3064	NA	NA	6,982 ± 6,025	97,433 (56,948)

Barcode03	MIP2461	NA	NA	14,178 ± 16,934	222,956 (125,873)
Barcode04	CD3406	NA	NA	13,999 ± 15,835	189,643 (151,880)
Barcode05	SAF3325	NA	NA	4,018 ± 3,404	179,637 (79,730)
Barcode06	SF1671	NA	NA	13,740 ± 14,671	192,841 (126,382)
Barcode07	MIP2602	NA	NA	8,263 ± 10,441	150,931 (109,403)
Barcode08	HIS2824	NA	NA	6,356 ± 8,126	227,134 (105,222)
Barcode09	SF1039	NA	NA	3,152 ± 5,513	137,029 (84,785)
Barcode10	MIP2422	NA	NA	9,581 ± 11,334	172,762 (133,652)
Barcode11	MIP2473	NA	NA	12,602 ± 15,327	227,602 (137,709)
Barcode12	SAF3333	NA	NA	2,689 ± 4,408	131,075 (102,127)

Basecalled and mapped mean and maximum read length in base pair per barcode and library. Basecalled values were obtained from Guppy's report (i.e. lengths of reads for which a barcode was recovered). Mapped values were obtained from Minimap2 report (i.e. reads that mapped to curated genomes). Mean length is reported with its standard deviation. Maximum length is also reported in parenthesis with the actual maximum nucleotide length matched to curated genomes by Minimap2 since reads may only be partially mappable due to the presence of artifactual sections (see methods and Table 5). Note that in PCR, the mean length of reads classified by mapping with Minimap2 always exceeds mean length of reads classified by Guppy because numerous reads classified by Guppy are short and unmapped (artifactual of very low quality reads).

Fig. S3. Cumulative read lengths profiles

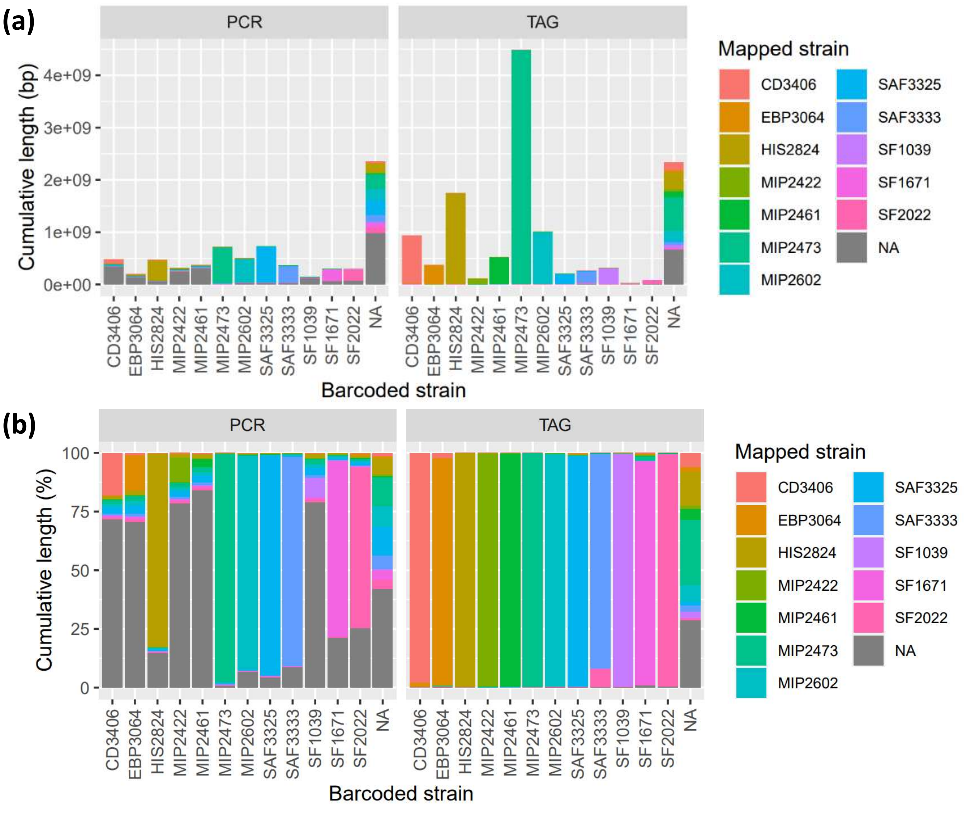

Taxonomic profile in terms of cumulative read lengths per barcoded strain for barcoded run PCR and TAG in (a) base pair or (b) percentage of total base pair. Note the abundance of unmapped reads in the PCR runs as compared to TAG (as « NA » in the legend, representing artifactual of very low quality reads). Note as well the presence of reads leaked from SF2022 in SAF3333 for TAG. Leakage may also occur in PCR but the common presence in b of multiple strains (multiple colors) on most strains profile suggest that cross mapping of short nanopore reads may be responsible for the pattern.
