# Supplementary material for: A comparison of Oxford nanopore library strategies for bacterial genomics
Source: BMC Genomics. 2023 Oct 20;24:627. doi: 10.1186/s12864-023-09729-z (PMC10589936; doi:10.1186/s12864-023-09729-z)
Supplement: Supplementary file 5 — Additional file 5. DNA extraction protocol. [file 12864_2023_9729_MOESM5_ESM.pdf]

Modified protocol Wizard® Genomic DNA Purification Kit from Promega

Produce overnight culture of isolated strains in 10mL
Pellet 2mL by centrifugation for 5 min at 13,000 rpm [1]
Resuspend the pellet in 540 µL of EDTA at 50 mM, ph 8.5 and 60 µL of lysozyme at 10 mg/mL
Incubate 1 h 30 at 37°C
Centrifuge for 3 min at 13,000 rpm
DISCARD the supernatant
Resuspend the pellet in 600 µL of 'Nucleic Acid Solution' (from kit) and mix
Heat for 5 min at 80°C (allow cooling off until next step)
Add 3 µL of RNAse (from kit)
Incubate 1 h 00 at 37°C
Add 200 µL of 'Protein Precipitation Solution' (from kit)
Vortex and place on ice for 5 min
Centrifuge for 3 min at 13,000 rpm
TRANSFER supernatant to a 1.5 mL tube
Add 600 µL isopropanol at ambient temperature
Mix by inverting the tube
Centrifuge for 3 min at 13,000 rpm
DISCARD the supernatant[2]
Add 600 µL of 70% ethanol at ambient temperature
Centrifuge for 3 min at 13,000 rpm[3]
DISCARD ethanol
Dry pellet at 37°C
Resuspend gDNA in 50-100 µL of water or elution buffer (from kit)

Notes :
[1]Pellet more than one tube per culture if more DNA is necessary. These can be eluted in smaller volumes and pooled at the elution step. Extracting 1 to 4 pellets may be necessary depending on the bacterial strain to achieve desired DNA amounts.
[2]The DNA pellet may not always be visible. The ethanol wash usually reveals it a bit more
[3]Centrifuge longer and faster if the white pellet remains loose in order to facilitate collecting clean supernatant.

QC

Assess DNA integrity on 2% gel electrophoresis (1-2 µL DNA + 4 µL loading DYE).
Measure the final concentration on Qubit (broad range kit).
Pool, dilute (with H2O) and/or concentrate (with magnetic beads) extracts to necessary DNA amount & concentration.
